# Genome-wide CRISPR screening reveals ADCK3 as a key regulator in sensitizing endometrial carcinoma cells to MPA therapy

**DOI:** 10.1038/s41416-023-02347-2

**Published:** 2023-07-04

**Authors:** Zijing Zhang, Meng Zhang, Jingyi Zhou, Donglai Wang

**Affiliations:** 1 State Key Laboratory of Common Mechanism Research for Major Diseases & Department of Medical Genetics, Institute of Basic Medical Sciences & School of Basic Medicine, Chinese Academy of Medical Sciences & Peking Union Medical College, 100005 Beijing, China; 2 Department of Obstetrics and Gynecology, Peking University People’s Hospital, 100044 Beijing, China

**Keywords:** Endometrial cancer, Molecular medicine

## Abstract

**Background:**

The effectiveness of conservative treatment of endometrial carcinoma (EC) with oral progesterone therapy, such as medroxyprogesterone acetate (MPA), can be blunted due to primary or acquired resistance, but the underlying mechanisms remain incompletely defined.

**Methods:**

Genome-wide CRISPR screening was performed to identify potential regulators in response to MPA in Ishikawa cells. Crystal violet staining, RT–qPCR, western blotting, ChIP–qPCR and luciferase assays were employed to elucidate the p53-AarF domain-containing kinase 3 (ADCK3) regulatory axis and its roles in sensitizing EC cells to MPA treatment.

**Results:**

ADCK3 is identified as a previously unrecognized regulator in response to MPA in EC cells. Loss of ADCK3 in EC cells markedly alleviated MPA-induced cell death. Mechanistically, loss of ADCK3 primarily suppresses MPA-mediated ferroptosis by abrogating arachidonate 15-lipoxygenase (ALOX15) transcriptional activation. Moreover, we validated ADCK3 as a direct downstream target of the tumor suppressor p53 in EC cells. By stimulating the p53-ADCK3 axis, the small-molecule compound Nutlin3A synergized with MPA to efficiently inhibit EC cell growth.

**Conclusions:**

Our findings reveal ADCK3 as a key regulator of EC cells in response to MPA and shed light on a potential strategy for conservative EC treatment by activating the p53-ADCK3 axis to sensitize MPA-mediated cell death.

## Background

EC is one of the most common gynecological malignancies, and it ranks third in incidence among female cancers [1]. EC accounts for nearly 5% of female cancer cases and over 2% of cancer-related deaths worldwide in women [2]; the lifetime risk of EC in women is ~3%, and the median age at diagnosis is ~61 years old [3]. According to global cancer statistics, it is estimated that there were 417,000 newly diagnosed cases and nearly 97,000 deaths from EC in 2020 [1]. Epidemiological evidence has indicated that EC exhibited a steady increasing tendency in age-standardized incidence in recent decades [4], and more importantly, the incidence of EC is expected to continue to increase over the next decade [5].

Although surgical treatment provides a favorable prognosis for patients with early-stage EC, it is not ideal for young women who wish to preserve their fertility [6]. Oral progesterone therapy, such as MPA, represents an alternative strategy for conservative treatment of stage IA, low-grade EC patients or patients in advanced stages who cannot tolerate surgery [7, 8]. Although 50–70% of patients respond well to high-dose progesterone initially, de novo or acquired resistance is still a major problem during conservative EC treatment [9–11]. Dysfunction of progesterone receptor (PR) is thought to largely account for progesterone resistance [12]. In clinical practice, constant stimulation of progesterone may reduce PRB levels, which, in turn, results in an imbalance between PR- and estrogen receptor (ER)-mediated signaling and consequently leads to EC cell growth and invasion [12]. The aberrant expression of PR can be achieved through epigenetic mechanisms [13]. Evidence has shown that epigenetic modulators can upregulate functional PR and restore the sensitivity of EC to progesterone therapy [14]. Additionally, the metabolic alterations and redox imbalance upon EC onset are also highly relevant to progesterone resistance, and multiple molecular disorders are involved [15–18]. However, due to the complexity of progesterone-mediated conservative treatment of EC, the regulators and molecular mechanisms governing progesterone resistance in EC cells remain incompletely defined.

ADCK3 (also known as COQ8A) is a mitochondrial protein that acts as an atypical kinase involved in the biosynthesis of coenzyme Q (CoQ) [19]. Based on its structural features, ADCK3 is assumed to play a regulatory, rather than a catalytic, role during CoQ biosynthesis [19]. Recently, ADCK3 was reported to possess ATPase activity that is responsible for the integrity of the CoQ biosynthesis-related protein complex [20, 21]. Mutations in *ADCK3* are associated with the development of progressive neurological disorders caused by primary CoQ deficiency due to ADCK3 dysfunction [22–24]. Mechanistically, decreased CoQ levels in ADCK3-deficient cells lead to impaired sulfur oxidation pathways, resulting in sulfide accumulation and consequent increased oxidative stress [25]. Replenishment of exogenous CoQ may partially rescue CoQ-dependent enzymatic activity, ATP production, and cellular levels of oxygen-free radicals in the fibroblasts of patients with *ADCK3* mutations [26, 27]. To date, the potential roles of ADCK3 in tumorigenesis or tumor progression have been poorly characterized. Whether and how ADCK3 participates in regulating progesterone resistance during conservative EC treatment remain largely unknown.

The RNA-guided CRISPR/Cas9 can be programmed to induce DNA double-strand breaks (DSBs), resulting in frameshift indel or premature termination codon mutations at specific genomic loci [28]. CRISPR/Cas9 has been combined with genome-scale guide RNA libraries to investigate gene function in a genome-wide manner and elucidate genotype–phenotype interactions in terms of cell proliferation, immunotherapy and drug resistance in human diseases, including cancer [29].

In this study, we performed genome-wide CRISPR knockout screening to uncover potential factors involved in regulating EC cell responsiveness to MPA therapy. Among these identified candidates, we confirmed that loss of ADCK3 rendered EC cells resistant to MPA. Loss of ADCK3 repressed MPA-induced ferroptosis through transcriptional downregulation of ALOX15. Interestingly, we validated that ADCK3 was a direct downstream target of p53 in EC cells. Combined therapy with Nutlin3A and MPA synergistically suppressed EC cell growth. This effect was achieved partially through Nutlin3A-induced activation of the p53-ADCK3 axis, which, in turn, sensitized EC cells to MPA-induced cell death.

## Methods

### Cell culture, constructs, transfection, and reagents

The Ishikawa, HEK293T and H1299 cell lines were cultured in DMEM (Corning, 10-013-CVR) supplemented with 10% (v/v) FBS (Gibco, 10099141). The HEC-265 cell line was cultured in EMEM (M&C Gene Technology, CM10010) with 15% FBS. The AN3CA cell line was cultured in EMEM supplemented with 10% FBS, 1× NEAA (Gibco, 11140050) and 1 mM sodium pyruvate (M&C Gene Technology, CC007). The cell lines were originally purchased from ATCC or Cell Resource Center of IBMS-CAMS, freshly thawed from our stock and cultured for no longer than 2 months. All cell lines were negative for mycoplasma contamination. The transfections were conducted by using Lipofectamine 2000 (Invitrogen, 11668500) according to the manufacturer’s protocol. To generate a luciferase reporter, annealed oligos were cloned into a pGL3-basic vector (Promega, E1761). MPA was purchased from Selleck (S2567). The following reagents were used as: Z-VAD-FMK (Solarbio, IZ0050) 10 µg/ml; necrostatin-1 (Sigma‒Aldrich, N9037) 10 µg/ml; ferrostatin-1 (Sigma‒Aldrich, SML0583) 2 µM; 3-MA (Sigma‒Aldrich, M9281) 2 mM; PD146176 (Selleck, S6956) 5 µM; liproxstatin-1 (Selleck, S7699) 2 µM; UAMC-3203 (Selleck, S8792) 2 µM. The antibodies and the sequence of siRNA, sgRNA and primers are listed in Supplementary Table S1.

### CRISPR screening

In total, 400 ng human Brunello library (Addgene, 73178) was transformed into 100 μl MegaX DH10B T1R Electrocomp™ Cells (Invitrogen, C6400-03) for electroporation, and then the cells were seeded into 15-cm dishes and incubated in a shaker incubator for 11–14 h at 32 °C, 220 rpm. The plasmid DNA was extracted with an EndoFree Plasmid Maxi Kit (Qiagen, 12362). The lentivirus was produced by lipofectamine-mediated transfection of Brunello library and packaging plasmids in HEK293T cells cultured in UltraCULTURE^TM^ Serum-free Medium (Lonza, BEBP12-725F). The viruses were harvested every 24 h for next 3 days. A total of 2 × 10^7^ Ishikawa cells were infected with lentivirus (MOI = 0.3) for 48 h and further selected with puromycin for 2 days. The selected cells were grown in regular medium for 7 days, and then treated with 40 µM MPA for 4 days. After that, the cells were cultured in regular medium for additional 10 days. gDNA from each cell group was isolated using a Blood&Cell Culture DNA Maxi Kit (Qiagen, 13362) and amplified by PCR. The PCR products were purified and subjected to NGS by using the Novaseq 6000-PE150 platform (Novogene, Beijing).

### Generation of ADCK3-KO cells

The oligos producing sgRNA to target *ADCK3* were annealed and cloned into the BbsI-linearized lentiCRISPR v2 construct (Addgene, 52961). The lentivirus expressing Cas9 and the indicated sgRNA were collected from HEK293T cells transfected with the expression construct and the packaging constructs, including pMD2.G (Addgene, 12259) and psPAX2 (Addgene 12260). Ishikawa cells were infected with the lentivirus and subjected to selection with puromycin (1 μg/ml; Invitrogen, A1113803). Two positive clones that were confirmed by western blot and sequencing were used for subsequent experiments.

### RT–qPCR

Total RNA was extracted from cultured cells using TRIzol reagent (Invitrogen, 15596018). cDNA was synthesized from 1 μg RNA using iScript™ Reverse Transcription Supermix (Bio-Rad, 1708841) according to the manufacturer’s protocol. The relative expression of each gene was measured in a Bio-Rad CFX Connect Real-Time PCR System by using the SYBR Green method (Tiangen, FP205-02). The expression of each target gene was normalized to *β-Actin* or *B2M*.

### Western blotting

Cells were harvested and lysed in NP-40 buffer with 1× protease inhibitor (Sigma–Aldrich, P8340) for 30 min on ice. Cell lysates were centrifuged at 15,000 rpm for 15 min at 4 °C, and the supernatant was collected. Equal amounts of protein from each sample denatured with 1× loading buffer were separated by SDS–PAGE, transferred to nitrocellulose membranes, recognized with indicated antibodies and exposed with ECL substrate (Pierce, 32106 or 34076).

### Luciferase assay

A firefly reporter containing wild-type or mutated p53-binding elements of the *ADCK3* promoter and a Renilla control reporter were cotransfected with or without p53-expressing constructs for 24 h, followed with or without indicated treatment for an additional 24 h. The relative luciferase activity was measured by the Dual-Luciferase Reporter Assay System (Promega, E1910) according to the manufacturer’s protocol.

### ChIP‒qPCR

Cells were fixed with 1% formaldehyde for 10 min at RT and lysed in ChIP Lysis Buffer for 10 min at 4 °C. After sonication, the lysates were centrifuged at 15,000 rpm for 10 min at 4 °C, and the supernatants were collected and precleaned in dilution buffer with 1× protease inhibitor incubated with salmon sperm DNA–saturated protein A agarose (Millipore, 16-157) for 1 h at 4 °C. The precleaned lysates were aliquoted equally and incubated with the indicated antibodies overnight at 4 °C. Then, each sample was incubated with saturated Protein A agarose for 2 h at 4 °C. After incubation, the agarose was washed sequentially with TSE I, TSE II, Buffer III and Buffer TE. The agarose-binding complex was eluted by elution buffer and reverse cross-linked for at least 6 h at 65 °C. Purified DNA was extracted by a PCR purification kit (QIAGEN, 28106), and qPCR was performed to detect the relative enrichment of the indicated transcription factor.

### In vitro cell proliferation assay

Cells were seeded at a density of 3 × 10^5^ into six-well plates with three replicates and cultured for three consecutive days. Then, the cell number was analyzed by crystal violet staining. The relative cell number was calculated by measuring the extracted crystal violet absorption at 590 nm.

### Xenograft tumor growth

A total of 1 × 10^7^ living cells were mixed with Matrigel (Corning, 354248) at a 2:1 ratio for a total volume of 200 μl. The cell-Matrigel mixture was then subcutaneously injected into B-NDG mice (NOD-*Prkdc*^*scid*^*Il2rg*^*tm1*^/Bcgen; 6 weeks old, female; Biocytogen). When the tumors grew to an average of 100 mm^3^, MPA (12 mg/kg) [30] and/or Nutlin3A (25 mg/kg) [31, 32] were administered daily by intraperitoneal injection. Approximately 2 weeks after treatment, the mice were sacrificed, and the tumor weight was measured.

### Lipid ROS measurement

The cells treated with or without MPA for 36 h were then incubated with C11-BODIPY staining solution (10 µM; ABclonal Technology, RM02821) in PBS for 20 min at 37 °C in the dark. The cells were rinsed twice with PBS and immediately analyzed by flow cytometry.

### Correlation analysis

GSE121367 dataset containing RNA-seq information of parental or acquired MPA-resistant Ishikawa cells was re-analyzed for the expression of ADCK3. The correlation of ADCK3 expression with MPA resistance was analyzed based on TCGA dataset. Specifically, a total of 6056 DEGs were identified in the GSE121367 dataset using “limma” analysis, filtered by |log_2_FC | >1, adjusted *P* value < 0.05. The 2975 upregulated genes were used as signatures of MPA resistance activity. ssGSEA was used to calculate MPA resistance activity in 548 EC samples of TCGA. Pearson analysis was used to calculate the correlation of ADCK3 expression with MPA resistance activity. “ggplot2” and “ggpubr” were used for visualization.

### Statistical analysis

The results are presented as the means ± SD. The difference was determined using a two-tailed, unpaired Student’s *t* test, one-way or two-way ANOVA, and PERMANOVA. *P* < 0.05 was denoted as statistically significant.

## Results

### Design of CRISPR screening for MPA resistance-related genes

The Brunello library used in this study is an optimized CRISPR knockout sgRNA library that is characterized by improved on-target and reduced off-target activity toward the human genome [33]. In general, the Brunello library comprises 4 unique sgRNAs for each of 19,114 genes along with 1000 nontargeting control sgRNAs [33]. We first expanded the library and assessed the distribution of sgRNA in the amplified library by next-generation sequencing. The histogram of sgRNA reads represents a great evenness of the library after amplification (Supplementary Fig. S1A). In addition, a scatter plot of sgRNA reads between the Brunello library and the sgRNA pool collected from the infected cells after puromycin selection showed that the complexity of the Brunello library was well maintained during the infection and selection processes (Supplementary Fig. S1B). In summary, the representation of our prepared sgRNA library was validated for qualifying the following screening applications.

**Fig. 1:**
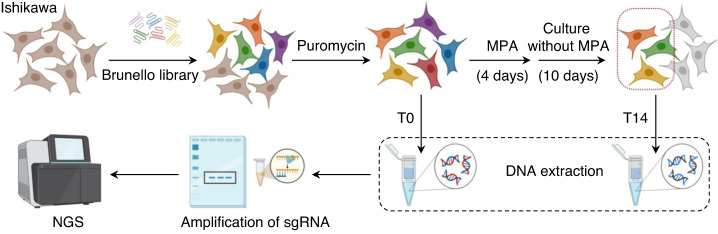
Design of CRISPR screening for MPA resistance-related genes. The pipeline of genome-wide CRISPR screening for Ishikawa cells in response to MPA treatment.

We sought to identify novel regulators involved in MPA-induced EC cell death by performing genome-wide CRISPR screening with the highly optimized Brunello CRISPR sgRNA library (Fig. 1). To this aim, we employed Ishikawa cells, a human EC cell line, as model cells since Ishikawa cells highly express PR [34]. We adopted a positive selection strategy in which the genes whose loss allowed cell survival in the presence of MPA were screened (Fig. 1). Specifically, Ishikawa cells successfully transduced with the Brunello sgRNA library and selected with puromycin were treated with MPA for 4 days, and the cells were allowed to grow and proliferation in MPA-null culture conditions for an additional 10 days (T14). The cells subjected to infection and selection but not treatment with MPA were set up as control cells (T0). Deep sequencing was performed to compare the abundance of all sgRNAs between the initial pooled cells (T0 cells) and MPA-treated pooled cells (T14 cells) (Fig. 1).

### CRISPR screening of the candidates in regulating MPA responsiveness

**Fig. 2:**
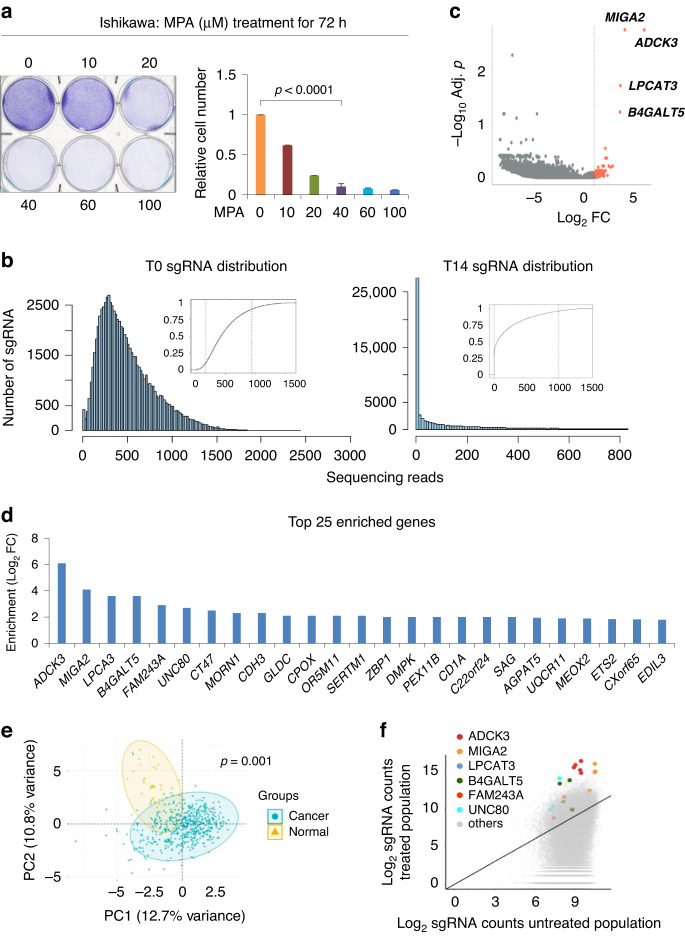
CRISPR screening of candidates involved in regulating MPA responsiveness. **a** Cell growth assay of Ishikawa cells treated with increasing concentrations of MPA, as indicated, for 72 h. **b** Histograms of sgRNA distribution in library-transduced Ishikawa cells with or without 40 μM MPA treatment. The initial condition after virus infection as a control (T0), a cell population after 4 days for MPA treatment and 10 days for normal culture post infection as treatment (T14). Inset: cumulative distribution of sequencing reads. The number of sequencing reads for the 10th and 90th sgRNA percentiles is indicated by the dashed red lines. **c** Volcano diagram of the genes enriched in living Ishikawa cells after MPA treatment during the screening. Red dots represent genes enriched for strong positive selection. Gray dots represent genes that are depleted for negative selection or not affected by MPA. **d** Top 25 candidate genes involved in MPA resistance regulation, identified by CRISPR screening. **e** Principal component analysis (PCA) was performed on the top 25 candidate genes identified by CRISPR screening. Each point represents an individual patient with endometrioid carcinoma or paracancerous tissues. **f** Scatter plot of sgRNA distribution of the top six candidate genes between the untreated population and MPA-treated population. Data are shown as the mean ± SD, *n* = 3.

To enrich the genes that are extremely critical in the regulation of MPA resistance during our screening, we treated Ishikawa cells with 40 μM MPA, a relatively high concentration of MPA used in vitro that dramatically reduced cell viability up to 80% in comparison with the control cells without MPA treatment (Fig. 2a). As shown in Fig. 2b (left panel), the histogram of sgRNA read counts from T0 cells revealed a great evenness of sgRNA distribution with a low number of missing sgRNAs, indicating good quality of cell preparation during the infection and selection processes. In contrast, MPA treatment resulted in an obviously heterogeneous distribution of sgRNA read counts across the target genes (Fig. 2b, right panel), indicating that the majority of sgRNA was lost due to MPA overselection-mediated cell death during our screening. Compared with T0 cells, 662 sgRNAs that corresponded to 166 genes were specifically enriched in T14 cells (Fig. 2c and Supplementary Tables S2 and S3). We noted the top 25 candidate genes whose enrichment levels were relatively higher than the others (Fig. 2d) since they could likely be more important in mediating MPA resistance than other less enriched genes. Interestingly, principal component analysis (PCA) of the gene expressional profiles of these top 25 screened candidate genes in EC samples from the TCGA dataset showed a dramatic discrepancy between the normal and malignant groups (Fig. 2e), further supporting that dysfunction of such candidate genes is critically involved in EC onset. In addition, we noticed that the mutation rate of these candidate genes, such as *ADCK3*, *B4GALT5*, *LPCAT3* and *UNC80*, was much lower than that of classical oncogenes (e.g., *PIK3CA*) or tumor suppression genes (e.g., *PTEN*), further prompting us to speculate that expression changes in these candidate genes could be a major mechanism involved in EC initiation or progression (Supplementary Fig. S2). Along with this notion, we paid much attention to *ADCK3* since *ADCK3* was the most enriched candidate and the four sgRNAs showed similar effects during screening (Fig. 2f), suggesting that downregulation of ADCK3 is most likely to render EC cells resistant to MPA.

### ADCK3 is critical for sensitizing EC cells to MPA treatment

**Fig. 3:**
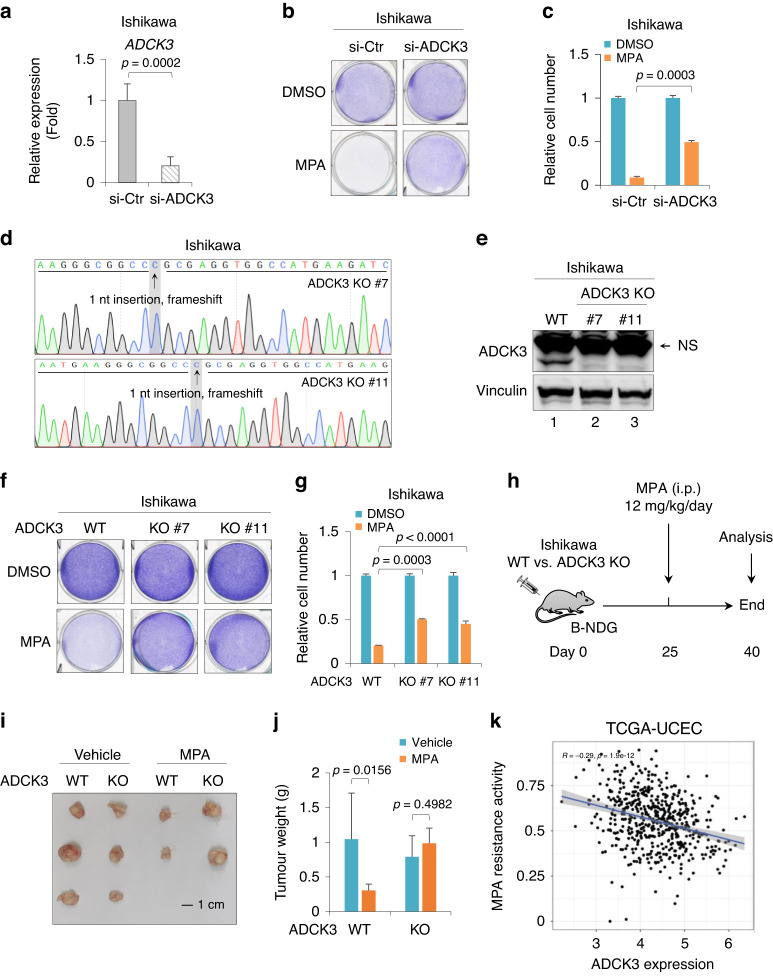
ADCK3 is critical to sensitize EC cells to MPA treatment. **a** RT–qPCR analysis of *ADCK3* expression in Ishikawa cells transfected with control siRNA or siRNA targeting ADCK3. **b** Cell growth assay by crystal violet staining of Ishikawa cells with or without ADCK3-KD in response to 40 μM MPA treatment for 3 days. **c** Quantitative analysis of the cell number in (**b**). **d** Sequencing analysis of *ADCK3* genomic DNA derived from the ADCK3-KO #7 and #11 cell lines. **e** WB analysis of ADCK3 in parental Ishikawa cells or ADCK3-KO single clones (#7 and #11). **f** Cell growth assay by crystal violet staining of parental or ADCK3-KO Ishikawa cells treated with or without 40 μM MPA for 3 days. **g** Quantitative analysis of the cell number in (**f**). **h** Working flow of the subcutaneous xenograft tumor growth model. **i** Xenograft tumors derived from Ishikawa cells with or without ADCK3 knockout, in response to MPA treatment. **j** Tumor weight analysis in (**i**). **k** Correlation analysis between *ADCK3* expression and MPA resistance in EC patients collected in TCGA-UCEC dataset. Data are shown as the mean ± SD, *n* = 3 in (**a**, **c**, **g**); *n* = 2 or 3 in (**j**), as indicated.

ADCK3 was considered an atypical protein kinase involved in CoQ biosynthesis [19]. However, the biological functions of ADCK3 in response to MPA-mediated conservative therapy for EC are unclear. We depleted endogenous ADCK3 by siRNA in Ishikawa cells, and the knockdown (KD) efficiency was confirmed by RT–qPCR (Fig. 3a). ADCK3-KD alone had no obvious effect on cell growth (Fig. 3b). However, MPA-induced cell death was markedly alleviated upon ADCK3-KD (Fig. 3b, c). In addition, we also evaluated other candidate genes. As shown in Supplementary Fig. S3A, MIGA2, LPCAT3 and UNC80 were efficiently depleted in Ishikawa cells. However, to our surprise, knockdown of these candidate genes individually showed minor rescue of MPA-induced cell death (Supplementary Fig. S3B, C). These results may reflect a functional complementation of these three candidate genes during MPA-induced cell death.

To further validate the effect of ADCK3 on MPA-induced cell death, we generated Ishikawa ADCK3 knockout (KO) cells by the CRISPR/Cas9 technique. We successfully obtained two independent KO clones, as confirmed by western blotting and DNA sequencing (Fig. 3d, e). Consistently, both single clonal cell lines with ADCK3 deletion exhibited obvious resistance to MPA treatment compared with parental cells (Fig. 3f, g). Since we used a relatively high concentration of MPA (40 μM) that was expected to display harsh cytotoxicity to EC cells, we then investigated the effect of ADCK3 on cell death in response to a low dosage of MPA. Treatment of parental Ishikawa cells with a low concentration of MPA (20 or 30 μM) also induced marked cell death (Supplementary Fig. S4A, B). More importantly, under such conditions, ADCK3-KO cells exhibited much stronger resistance to MPA-induced cell death, especially in response to 20 μM MPA treatment, where MPA-induced cell death was almost completely abrogated by ADCK3-KO (Supplementary Fig. S4A, B).

To measure whether ADCK3-mediated regulation of EC cell sensitivity to MPA is a general phenotype, we employed another PR-positive EC cell line, HEC-265, for testing. The KD efficiency of ADCK3 was confirmed in HEC-265 cells (Supplementary Fig. S4C). Similar to the results in Ishikawa cells, depletion of ADCK3 in HEC-265 cells also led to obvious rescue of MPA-induced cell death (Supplementary Fig. S4D, E). Progressive loss of PR expression in EC cells may confer acquired MPA resistance during conservative EC treatment in clinical practice [12], suggesting that PR is required for EC cells in response to MPA. To evaluate whether the effect of ADCK3 on cell death upon MPA treatment is reliant on the presence of PR, we knocked down ADCK3 in AN3CA cells, a PR-negative EC cell line (Supplementary Fig. S4F). We found that AN3CA cells were highly resistant to MPA treatment, even at the relatively high concentration used in this assay (40 μM) (Supplementary Fig. S4G, H). Accordingly, depletion of ADCK3 showed no effect on MPA-induced cell death (Supplementary Fig. S4G, H), supporting that PR is required for ADCK3-mediated sensitization of EC cells to MPA.

To corroborate our findings in vivo, we established a xenograft tumor growth model to observe potential MPA-mediated regression of tumor growth (Fig. 3h). As shown in Fig. 3i, j, MPA treatment inhibited the tumor growth of parental Ishikawa cells. However, the tumor growth of Ishikawa cells with ADCK3-KO exhibited no obvious responsiveness to MPA treatment (Fig. 3i, j). Furthermore, by analyzing the TCGA-UCEC dataset, we observed that the low expression of ADCK3 was significantly correlated with MPA resistance (Fig. 3k). Long-term treatment with a low dosage of MPA can induce acquired MPA resistance in EC cells [12]. Interestingly, by analyzing the dataset containing gene expressional profiles of parental Ishikawa cells and Ishikawa cells with acquired MPA resistance [35], we found that *ADCK3* expression was reduced in MPA-resistant Ishikawa cells (Supplementary Fig. S4I), suggesting that ADCK3 downregulation might also be relevant to acquired MPA resistance in EC cells. Taken together, our data indicate that ADCK3 is critically involved in sensitizing EC cells to MPA treatment.

### ADCK3 contributes to MPA-induced ferroptosis by upregulating ALOX15

**Fig. 4:**
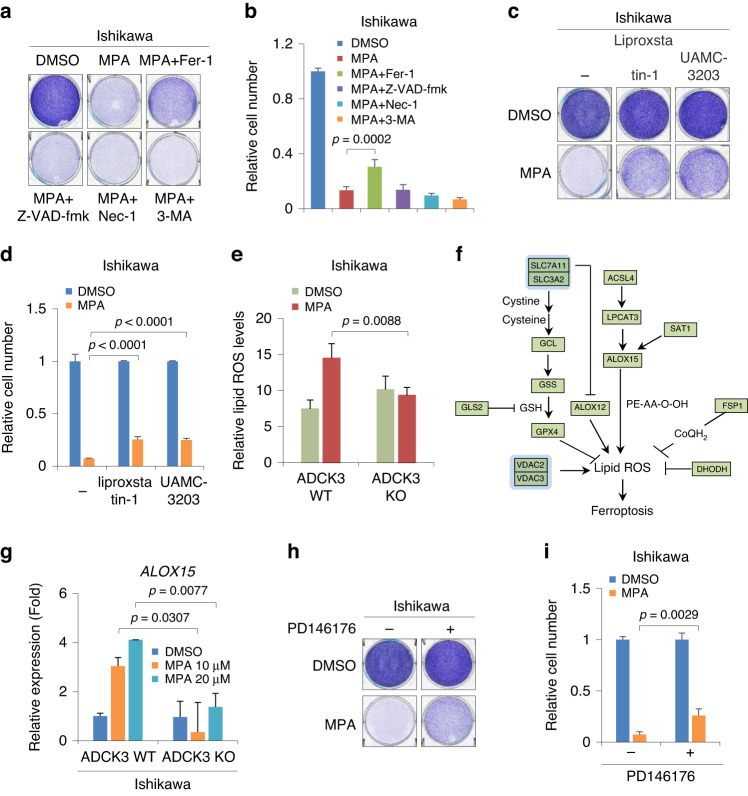
ADCK3 contributes to MPA-induced ferroptosis by upregulating ALOX15. **a** Cell growth assay by crystal violet staining of Ishikawa cells treated with 40 μM MPA for 3 days in the presence or absence of Fer-1 (2 μM), Z-VAD-fmk (10 μg/ml), Nec-1 (10 μg/ml), or 3-MA (2 mM). **b** Quantitative analysis of the cell number in (**a**). **c** Cell growth assay by crystal violet staining of Ishikawa cells treated with 40 μM MPA for 3 days in the presence or absence of the ferroptosis inhibitor liproxstatin-1 (2 μM) or UAMC-3203 (2 μM). **d** Quantitative analysis of the cell number in (**c**). **e** Lipid ROS measurement by C11-BODIPY in parental or ADCK3-KO Ishikawa cells treated with or without 40 μM MPA for 36 h. **f** A schematic diagram of ferroptosis-related pathways. **g** RT–qPCR analysis of *ALOX15* expression in parental or ADCK3-KO Ishikawa cells treated with increasing concentrations of MPA, as indicated. **h** Cell growth assay by crystal violet staining of Ishikawa cells treated with 40 μM MPA for 3 days in the presence or absence of the ALOX15 inhibitor PD146176 (5 μM). **i** Quantitative analysis of the cell number in (**h**). Data are shown as the mean ± SD, *n* = 3 in (**b**, **d**, **e**, **i**); *n* = 2 in (**g**).

MPA may induce cell death in various ways [36–38]. To determine the underlying mechanism(s) by which the MPA-ADCK3 axis promotes EC cell death, we sought to inhibit the potential cell death pathways employed by MPA. To this end, Z-VAD-fmk, ferrostatin-1 (Fer-1), 3-methyladenine (3-MA) and necrostatin-1 (Nec-1) were used to block apoptosis, ferroptosis, autophagic cell death and necroptosis, respectively. As shown in Fig. 4a, b, the inhibition of the ferroptosis pathway by Fer-1 markedly, but not completely, blocked MPA-induced cell death in Ishikawa cells. In contrast, blockade of either apoptosis, autophagic cell death or the necroptosis pathway alone had a negligible effect on MPA-induced cell death (Fig. 4a, b). In addition, we monitored major cell death pathway(s) in response to MPA treatment in HEC-265 cells. Similarly, we noticed that only Fer-1 successfully alleviated MPA-induced cell death (Supplementary Fig. S5A, B). To exclude the potential bias of Fer-1 in terms of its efficacy among different types of tumor cells, we attempted to block the ferroptosis pathway by using distinct ferroptosis inhibitors, including liproxstatin-1 and UAMC-3203. Interestingly, both inhibitors rescued MPA-induced cell death to similar levels (Fig. 4c, d), and these levels were comparable to those after Fer-1 treatment (Fig. 4b vs. d). These results indicate that multiple cell death pathways may cooperate in MPA-induced cell death and that ferroptosis could be a major pathway involved in MPA-induced cell death in EC.

The accumulation of lipid reactive oxygen species (ROS) represents a hallmark feature of ferroptosis [39]. Indeed, we observed that MPA treatment elevated lipid ROS levels in parental Ishikawa cells (Fig. 4e). Although the basal levels of lipid ROS were slightly increased in ADCK3-KO cells, we still found that the abrogation of ADCK3 profoundly blocked MPA-induced lipid ROS production (Fig. 4e). These results suggested that loss of ADCK3 blunts MPA-induced ferroptosis of EC cells.

Ferroptotic cell death is a complex process that is modulated by different pathways involving numerous regulatory factors [39]. Therefore, we evaluated whether ADCK3 contributed to MPA-induced ferroptosis by regulating these key factors (Fig. 4f). To this end, we performed an RT–qPCR assay to detect their expression changes in the presence or absence of ADCK3 in response to MPA treatment. Among these factors, *ALOX15*, *SLC7A11* and *SLC3A2* had markedly increased expression in parental Ishikawa cells by MPA, and the effect was dose-dependent (Fig. 4g and Supplementary Fig. S6). Notably, the elevated expression of *ALOX15*, *SLC7A11* and *SLC3A2* in response to MPA treatment was almost completely abrogated upon ADCK3 knockout (Fig. 4g and Supplementary Fig. S6). SLC7A11 and SLC3A2 form a heterodimer that functionally serves as a cystine/glutamate antiporter system [40]. Since accumulated evidence indicates that blocking cysteine uptake by inhibition or downregulation of SLC7A11 triggers ferroptosis [41], we speculated that the upregulation of SLC7A11 and its functional partner SLC3A2 might not account for MPA-induced, ADCK3-involved ferroptosis. In contrast, ALOX15, an enzyme that oxidizes polyunsaturated fatty acids, has been reported to increase lipid ROS and participate in ferroptosis induction in cancer cells [42]. Therefore, our data suggest that ALOX15 is a key effector downstream of ADCK3 during MPA-induced ferroptosis in EC cells. To validate this point, we treated Ishikawa cells with PD146176, an ALOX15 inhibitor, to measure the potential changes in MPA-induced cell death. As shown in Fig. 4h, i, despite not completely rescued, inhibition of ALOX15 activity indeed markedly alleviated MPA-induced cell death. Taken together, our data reveal that ADCK3 contributes to MPA-induced ferroptosis at least in part by upregulating ALOX15.

### ADCK3 is a direct downstream target of p53 in EC cells

**Fig. 5:**
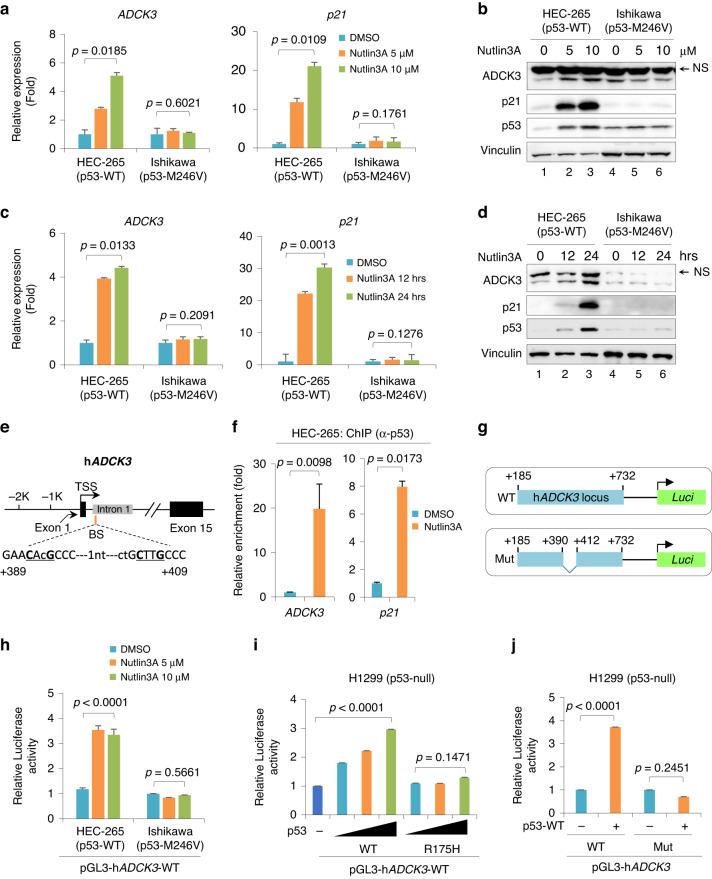
ADCK3 is a direct downstream target of p53 in EC cells. **a**, **b** RT–qPCR (**a**) or WB (**b**) analysis of ADCK3 expression in HEC-265 and Ishikawa cells, respectively, in response to increasing amounts of Nutlin3A, as indicated, for 24 h. The expression of the p53 classical downstream target p21 was used as a positive control. **c**, **d** RT–qPCR (**c**) or WB (**d**) analysis of ADCK3 expression in HEC-265 and Ishikawa cells, respectively, in response to 10 μM Nutlin3A for the indicated period of time, as indicated. The expression of the p53 classical downstream target p21 was used as a positive control. **e** Schematic diagram of human *ADCK3* (*hADCK3*) loci with potential p53-binding element. **f** ChIP‒qPCR analysis of p53 occupancy on *ADCK3* loci in HEC-265 cells treated with or without 10 μM Nutlin3A for 24 h. *p21* served as a positive control for p53 binding. **g** Schematic diagram of the *ADCK3* luciferase reporter with or without a putative p53-binding element (designated WT and Mut, respectively). **h** Luciferase assay of the WT *ADCK3* reporter in HEC-265 or Ishikawa cells treated with increasing amounts of Nutlin3A, as indicated, for 24 h. **i** Luciferase assay of the WT *ADCK3* reporter in p53-null H1299 cells transfected with increasing amounts of the wild-type or mutant p53-expressing construct, as indicated, for 24 h. **j** Luciferase assay of WT or Mut *ADCK3* reporter in H1299 cells transfected with wild-type p53-expressing construct for 24 h. Data are shown as the mean ± SD, *n* = 3 in (**h**, **i**, **j**); *n* = 2 in (**a**, **c**, **f**).

Since the presence of ADCK3 benefits EC cell sensitivity to MPA, we attempted to investigate how ADCK3 is regulated in EC cells. Interestingly, although ADCK3 is critical to MPA-induced cell death, MPA did not regulate ADCK3 levels in EC cells (Supplementary Fig. S7A, B). Previous literature showed that ADCK3 was upregulated in response to the MDM2 inhibitor Nutlin3A or DNA damage, and inhibition of ADCK3 expression partly suppressed p53-induced apoptosis [43]. Given that EC patients who receive conservative treatment with MPA are usually in stage IA, which has a relatively high percentage of tumors harboring wild-type p53 [44], we were then intrigued to evaluate whether p53 regulates ADCK3 in EC cells. As expected, Nutlin3A treatment easily upregulated *p21*, a canonical target gene of p53, in HEC-265 cells (conserved wild-type p53), but not in Ishikawa cells (conserved mutant p53, p53-M246V) (Fig. 5a). Under the same conditions, the expression of *ADCK3* was also elevated in response to increasing amounts of Nutlin3A in HEC-265 cells (Fig. 5a). Consistent with the changes in mRNA, the protein levels of both p21 and ADCK3 were gradually increased by Nutlin3A in a dose-dependent manner in HEC-265 cells but not in Ishikawa cells (Fig. 5b). Similarly, the upregulation of ADCK3 at both the mRNA and protein levels in HEC-265 cells was also observed in response to Nutlin3A treatment in a time-dependent manner (Fig. 5c, d). In summary, our data indicate that p53 transcriptionally regulates *ADCK3* in EC cells.

To investigate whether ADCK3 is a direct target of p53, we performed a ChIP assay to measure p53 binding to *ADCK3* loci. We noticed a potential p53-binding element located within intron 1 (+389 − +409) of *ADCK3* [45, 46], and p53 enrichment at this locus was significantly enhanced upon Nutlin3A treatment (Fig. 5e, f). To evaluate whether p53 binding to this element has a functional consequence, we conducted a series of luciferase assays with reporters containing the wild-type or mutant p53-binding element of *ADCK3* (Fig. 5g). As shown in Fig. 5h, Nutlin3A treatment increased the luciferase activity of the wild-type reporter in HEC-265 cells but not in Ishikawa cells. In addition, only wild-type p53, but not the transactivation-deficient p53-R175H mutant, was able to drive the expression of the *ADCK3* reporter containing the wild-type p53-binding element (Fig. 5i). In contrast, wild-type p53 failed to activate the luciferase reporter harboring a mutant p53-binding element (Fig. 5j). All these results revealed that p53 drives *ADCK3* transcriptional activation by binding with *ADCK3* loci. Taken together, our data identify *ADCK3* as a downstream target gene of p53 in EC cells.

### Activation of the p53-ADCK3 axis synergizes with MPA to suppress EC cell growth

**Fig. 6:**
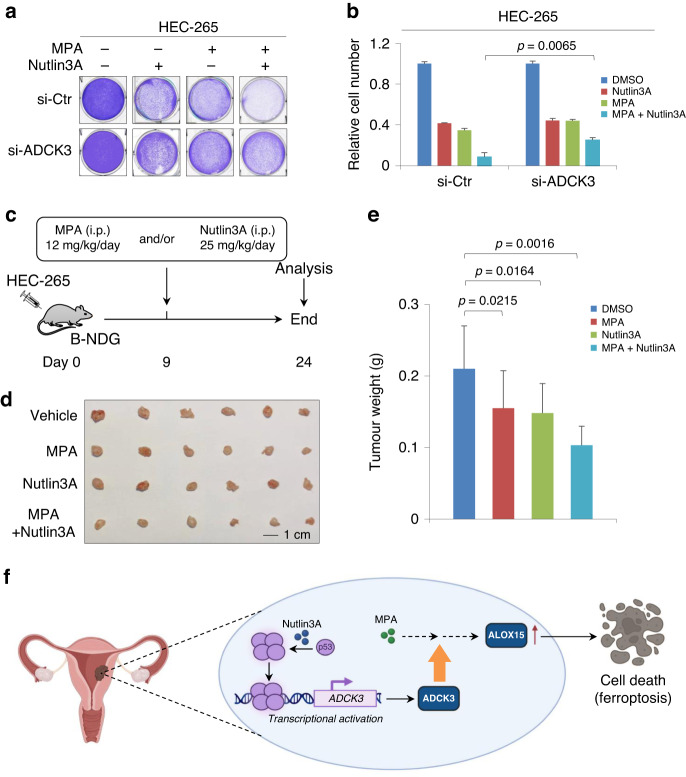
Synergistic effect of MPA and Nutlin3A on EC cell growth suppression. **a** Cell growth assay by crystal violet staining of control or ADCK3-KD HEC-265 cells treated individually or together with 20 μM MPA and 10 μM Nutlin3A, as indicated, for 3 days. **b** Quantitative analysis of the cell number in (**a**). **c** Working flow of the combination therapy analyses by using subcutaneous xenograft tumor growth model. **d** Xenograft tumor growth of HEC-265 cells in B-NDG mice under the indicated administration. **e** Tumor weight analysis in (**d**). **f** A working model of the synergistic effect of Nutlin3A and MPA on EC cell death through activation of the p53-ADCK3 axis and ALOX15-involved ferroptosis. Data are shown as the mean ± SD, *n* = 3 in (**b**); *n* = 6 in (**e**).

The maintenance of wild-type p53 in tumor tissues is a favorable marker for EC patients [47]. Therefore, we assessed whether p53-mediated ADCK3 activation enhances the effect of conservative EC treatment with MPA. Notably, MPA alone did not activate p53, as neither stabilization of p53 nor upregulation of p21 (a p53 downstream target) was observed in response to MPA treatment in HEC-265 cells (Supplementary Fig. S7B). To this aim, we stimulated the p53-ADCK3 axis with Nutlin3A and evaluated the potential synergistic effect of Nutlin3A and MPA on the growth suppression of EC cells. As shown in Fig. 6a, b, individual treatment with Nutlin3A or MPA exhibited a relatively modest antiproliferative effect on control HEC-265 cells, whereas the combination treatment with Nutlin3A and MPA displayed a much stronger suppressive effect on cell growth, confirming that Nutlin3A and MPA synergize to antagonize EC cell growth. To investigate whether ADCK3 contributes to such a synergistic effect, we depleted ADCK3 in HEC-265 cells and observed a reduced antiproliferative effect by combined treatment with Nutlin3A and MPA (Fig. 6a, b), suggesting that upregulation of ADCK3 by Nutlin3A in p53-wild-type cells is critically involved in enhancing MPA-induced EC cell growth inhibition.

To corroborate the synergistic effect on tumor suppression by combination therapy in vivo, we evaluated the combination treatment of MPA and Nutlin3A in a HEC-265 cell-based xenograft tumor model (Fig. 6c). As expected, treatment with MPA alone was able to inhibit tumor growth (Fig. 6d, e), although the suppressive effect on the growth of HEC-265 cells in vivo was relatively weak compared with that of the Ishikawa cell-based xenograft model (Fig. 3i, j). In addition, Nutlin3A treatment induced xenograft tumor growth regression (Fig. 6d, e). More importantly, the combination of MPA and Nutlin3A displayed a much more potent suppressive effect on tumor growth than either treatment alone (Fig. 6d, e). Taken together, our data reveal a potential therapeutic strategy for EC involving activation of the p53-ADCK3 axis, which, in turn, sensitizes EC cells to conservative treatment with MPA (Fig. 6f).

## Discussion

In this study, by employing genome-scale CRISPR screening, we identified and confirmed ADCK3 as a key regulator in MPA-induced EC cell death. During this screening, we adopted a positive selection strategy to specifically enrich the sgRNAs remaining in living EC cells after MPA treatment. However, we cannot exclude the possibility that certain genes targeted by sgRNAs in dead cells render EC cells more sensitive to MPA treatment. Therefore, a negative selection strategy could also be worthy of future research. In addition, we deliberately overselected the cells by using a high concentration of MPA (Fig. 2b) to discover candidates that are extremely important for impairing MPA resistance in EC cells. Interestingly, the candidates revealed by this strategy seemed to be highly associated with EC initiation or progression, since the top 25 candidates already worked well as markers to differentiate normal endometrial tissues and malignant tumors of EC (Fig. 2e).

Ferroptosis is a novel form of programmed cell death driven by iron-dependent lipid peroxidation [39]. A recent study showed that ferroptosis-related genes may play a crucial role in EC through multiple biological processes, including the p53 signaling pathway [48]. For example, p53 reduces the production of glutathione by transcriptionally decreasing SLC7A11 levels to hamper the function of GPX4, leading to lipid peroxidation and ferroptosis [39]. In addition, overexpression of spermidine/spermine N1-acetyltransferase 1 (SAT1), a direct p53 target gene, promotes ROS-induced ferroptosis by upregulating ALOX15 via unclear mechanisms [42]. In our study, we found that MPA elevated intracellular lipid ROS and induced ferroptosis in EC cells, largely in a manner dependent on the presence of ADCK3 (Fig. 4e). Given that we validated ADCK3 as a downstream target gene of p53 (Fig. 5a–j), our study revealed an alternative p53-ADCK3-ALOX15 axis in regulating ferroptosis, which could be complementary to the p53-SAT1-ALOX15 pathway in ferroptosis induction in response to lipid ROS. Furthermore, bioinformatic analyses have revealed that the expression of ferroptosis-related genes appears to predict prognosis and to be closely correlated with drug resistance in EC [49, 50]. Since evidence has shown a reduction in ALOX15 levels during the EC tumorigenesis process [51], more studies are needed to determine whether ALOX15 could be a potential prognostic biomarker of EC in the future.

The maintenance of wild-type p53 in EC tissues represents an important biomarker for patients with a favorable prognosis [47, 52]. In fact, the majority of EC patients who are candidates for conservative therapy with progesterone usually have stage IA and low-grade tumors, and these cases have a relatively high rate of wild-type p53 expression [44, 53, 54]. Therefore, it is reasonable to speculate that activation of the p53 signaling pathway could benefit progesterone-mediated conservative EC treatment. Indeed, our observations revealed that stimulation of p53 with the small-molecule compound Nutlin3A sensitized EC cells to MPA treatment in vitro and in vivo (Fig. 6a–e). This synergistic effect could be partially attributed to p53-mediated ADCK3 transcriptional upregulation in response to Nutlin3A, since ADCK3 was critically required for MPA-induced EC cell death (Fig. 3a–j). Taken together, our study provides mechanistic insights into how the tumor suppressor p53 facilitates conservative EC treatment and presents a potential strategy for enhancing MPA-induced conservative EC treatment by stimulating the p53-ADCK3 axis.

## Supplementary information

Supplementary Figures and Tables

## Data Availability

All data generated or analyzed during this study are included in this published article and its supplementary information files.

## References

[1] Sung H, Ferlay J, Siegel RL, Laversanne M, Soerjomataram I, Jemal A (2021). Global Cancer Statistics 2020: GLOBOCAN estimates of incidence and mortality worldwide for 36 cancers in 185 countries. CA: Cancer J Clin.

[2] Ferlay J, Soerjomataram I, Dikshit R, Eser S, Mathers C, Rebelo M (2015). Cancer incidence and mortality worldwide: sources, methods and major patterns in GLOBOCAN 2012. Int J Cancer.

[3] Crosbie EJ, Kitson SJ, McAlpine JN, Mukhopadhyay A, Powell ME, Singh N (2022). Endometrial cancer. Lance.

[4] Lortet-Tieulent J, Ferlay J, Bray F, Jemal A (2018). International patterns and trends in endometrial cancer incidence, 1978-2013. J Natl Cancer Inst.

[5] Sheikh MA, Althouse AD, Freese KE, Soisson S, Edwards RP, Welburn S (2014). USA endometrial cancer projections to 2030: should we be concerned?. Future Oncol.

[6] Kovacevic N (2021). Surgical treatment and fertility perservation in endometrial cancer. Radiol Oncol.

[7] Trojano G, Olivieri C, Tinelli R, Damiani GR, Pellegrino A, Cicinelli E (2019). Conservative treatment in early stage endometrial cancer: a review. Acta bio-medica : Atenei Parmensis.

[8] Jerzak KJ, Duska L, MacKay HJ (2019). Endocrine therapy in endometrial cancer: an old dog with new tricks. Gynecol Oncol.

[9] Ushijima K, Yahata H, Yoshikawa H, Konishi I, Yasugi T, Saito T (2007). Multicenter phase II study of fertility-sparing treatment with medroxyprogesterone acetate for endometrial carcinoma and atypical hyperplasia in young women. J Clin Oncol.

[10] Ramirez PT, Frumovitz M, Bodurka DC, Sun CC, Levenback C (2004). Hormonal therapy for the management of grade 1 endometrial adenocarcinoma: a literature review. Gynecol Oncol.

[11] Chiva L, Lapuente F, González-Cortijo L, Carballo N, García JF, Rojo A (2008). Sparing fertility in young patients with endometrial cancer. Gynecol Oncol.

[12] Zhao S, Li G, Yang L, Li L, Li H (2013). Response-specific progestin resistance in a newly characterized Ishikawa human endometrial cancer subcell line resulting from long-term exposure to medroxyprogesterone acetate. Oncol Lett.

[13] Xiong Y, Dowdy SC, Gonzalez Bosquet J, Zhao Y, Eberhardt NL, Podratz KC (2005). Epigenetic-mediated upregulation of progesterone receptor B gene in endometrial cancer cell lines. Gynecol Oncol.

[14] Ren Y, Liu X, Ma D, Feng Y, Zhong N (2007). Down-regulation of the progesterone receptor by the methylation of progesterone receptor gene in endometrial cancer cells. Cancer Genet Cytogenet.

[15] Eberlé D, Hegarty B, Bossard P, Ferré P, Foufelle F (2004). SREBP transcription factors: master regulators of lipid homeostasis. Biochimie..

[16] Ma X, Zhao T, Yan H, Guo K, Liu Z, Wei L (2021). Fatostatin reverses progesterone resistance by inhibiting the SREBP1-NF-κB pathway in endometrial carcinoma. Cell Death Dis.

[17] Ma X, Xia M, Wei L, Guo K, Sun R, Liu Y (2022). ABX-1431 inhibits the development of endometrial adenocarcinoma and reverses progesterone resistance by targeting MGLL. Cell Death Dis.

[18] Wang Y, Wang Y, Zhang Z, Park JY, Guo D, Liao H (2016). Mechanism of progestin resistance in endometrial precancer/cancer through Nrf2-AKR1C1 pathway. Oncotarget..

[19] Stefely JA, Reidenbach AG, Ulbrich A, Oruganty K, Floyd BJ, Jochem A (2015). Mitochondrial ADCK3 employs an atypical protein kinase-like fold to enable coenzyme Q biosynthesis. Mol Cell.

[20] Stefely JA, Licitra F, Laredj L, Reidenbach AG, Kemmerer ZA, Grangeray A (2016). Cerebellar ataxia and coenzyme Q deficiency through loss of unorthodox kinase activity. Mol Cell.

[21] Asquith CRM, Murray NH, Pagliarini DJ (2019). ADCK3/COQ8A: the choice target of the UbiB protein kinase-like family. Nat Rev Drug Discov.

[22] Hajjari M, Tahmasebi-Birgani M, Mohammadi-Asl J, Nasiri H, Kollaee A, Mahmoodi M (2019). Exome sequencing found a novel homozygous deletion in ADCK3 gene involved in autosomal recessive spinocerebellar ataxia. Gene..

[23] Sun M, Johnson AK, Nelakuditi V, Guidugli L, Fischer D, Arndt K (2019). Targeted exome analysis identifies the genetic basis of disease in over 50% of patients with a wide range of ataxia-related phenotypes. Genet Med.

[24] Horvath R, Czermin B, Gulati S, Demuth S, Houge G, Pyle A (2012). Adult-onset cerebellar ataxia due to mutations in CABC1/ADCK3. J Neurol Neurosurg Psychiatry.

[25] Ziosi M, Di Meo I, Kleiner G, Gao XH, Barca E, Sanchez-Quintero MJ (2017). Coenzyme Q deficiency causes impairment of the sulfide oxidation pathway. EMBO Mol Med.

[26] Cullen JK, Abdul Murad N, Yeo A, McKenzie M, Ward M, Chong KL (2016). AarF domain containing kinase 3 (ADCK3) mutant cells display signs of oxidative stress, defects in mitochondrial homeostasis and lysosomal accumulation. PLoS ONE.

[27] Shalata A, Edery M, Habib C, Genizi J, Mahroum M, Khalaily L (2019). Primary coenzyme Q deficiency due to novel ADCK3 variants, studies in fibroblasts and review of literature. Neurochem Res.

[28] Cong L, Ran FA, Cox D, Lin S, Barretto R, Habib N (2013). Multiplex genome engineering using CRISPR/Cas systems. Science.

[29] Shalem O, Sanjana NE, Hartenian E, Shi X, Scott DA, Mikkelson T (2014). Genome-scale CRISPR-Cas9 knockout screening in human cells. Science.

[30] Cui Y, Wu H, Yang L, Huang T, Li J, Gong X (2021). Chlorpromazine sensitizes progestin-resistant endometrial cancer cells to MPA by upregulating PRB. Front Oncol.

[31] Drakos E, Singh RR, Rassidakis GZ, Schlette E, Li J, Claret FX (2011). Activation of the p53 pathway by the MDM2 inhibitor nutlin-3a overcomes BCL2 overexpression in a preclinical model of diffuse large B-cell lymphoma associated with t(14;18)(q32;q21). Leukemia..

[32] Mouraret N, Marcos E, Abid S, Gary-Bobo G, Saker M, Houssaini A (2013). Activation of lung p53 by Nutlin-3a prevents and reverses experimental pulmonary hypertension. Circulation..

[33] Doench JG, Fusi N, Sullender M, Hegde M, Vaimberg EW, Donovan KF (2016). Optimized sgRNA design to maximize activity and minimize off-target effects of CRISPR-Cas9. Nat Biotechnol.

[34] Qu W, Zhao Y, Wang X, Qi Y, Zhou C, Hua Y (2019). Culture characters, genetic background, estrogen/progesterone receptor expression, and tumorigenic activities of frequently used sixteen endometrial cancer cell lines. Clinica chimica acta Int J Clin Chem.

[35] Li W, Wang S, Qiu C, Liu Z, Zhou Q, Kong D (2019). Comprehensive bioinformatics analysis of acquired progesterone resistance in endometrial cancer cell line. J Transl Med.

[36] Ohta K, Maruyama T, Uchida H, Ono M, Nagashima T, Arase T (2008). Glycodelin blocks progression to S phase and inhibits cell growth: a possible progesterone-induced regulator for endometrial epithelial cell growth. Mol Hum Reprod.

[37] Dai D, Wolf DM, Litman ES, White MJ, Leslie KK (2002). Progesterone inhibits human endometrial cancer cell growth and invasiveness: down-regulation of cellular adhesion molecules through progesterone B receptors. Cancer Res.

[38] Vereide AB, Kaino T, Sager G, Ørbo A (2005). Bcl-2, BAX, and apoptosis in endometrial hyperplasia after high dose gestagen therapy: a comparison of responses in patients treated with intrauterine levonorgestrel and systemic medroxyprogesterone. Gynecol Oncol.

[39] Li J, Cao F, Yin HL, Huang ZJ, Lin ZT, Mao N (2020). Ferroptosis: past, present and future. Cell Death Dis.

[40] Koppula P, Zhuang L, Gan B (2021). Cystine transporter SLC7A11/xCT in cancer: ferroptosis, nutrient dependency, and cancer therapy. Protein Cell.

[41] Jiang L, Kon N, Li T, Wang SJ, Su T, Hibshoosh H (2015). Ferroptosis as a p53-mediated activity during tumour suppression. Nature..

[42] Ou Y, Wang SJ, Li D, Chu B, Gu W (2016). Activation of SAT1 engages polyamine metabolism with p53-mediated ferroptotic responses. Proc Natl Acad Sci USA.

[43] Iiizumi M, Arakawa H, Mori T, Ando A, Nakamura Y (2002). Isolation of a novel gene, CABC1, encoding a mitochondrial protein that is highly homologous to yeast activity of bc1 complex. Cancer Res.

[44] Da Cruz Paula A, DeLair DF, Ferrando L, Fix DJ, Soslow RA, Park KJ (2021). Genetic and molecular subtype heterogeneity in newly diagnosed early- and advanced-stage endometrial cancer. Gynecol Oncol.

[45] Hafner A, Stewart-Ornstein J, Purvis JE, Forrester WC, Bulyk ML, Lahav G (2017). p53 pulses lead to distinct patterns of gene expression albeit similar DNA-binding dynamics. Nat Struct Mol Biol.

[46] Andrysik Z, Galbraith MD, Guarnieri AL, Zaccara S, Sullivan KD, Pandey A (2017). Identification of a core TP53 transcriptional program with highly distributed tumor suppressive activity. Genome Res.

[47] Köbel M, Ronnett BM, Singh N, Soslow RA, Gilks CB, McCluggage WG (2019). Interpretation of P53 immunohistochemistry in endometrial carcinomas: toward increased reproducibility. Int J Gynecol Pathol.

[48] Qin J, Shao X, Wu L, Du H (2021). Identification of the ferroptosis-associated gene signature to predict the prognostic status of endometrial carcinoma patients. Comput Math Methods Med.

[49] Weijiao Y, Fuchun L, Mengjie C, Xiaoqing Q, Hao L, Yuan L (2021). Immune infiltration and a ferroptosis-associated gene signature for predicting the prognosis of patients with endometrial cancer. Aging..

[50] He J, Ding H, Li H, Pan Z, Chen Q (2021). Intra-tumoral expression of SLC7A11 is associated with immune microenvironment, drug resistance, and prognosis in cancers: a pan-cancer analysis. Front Genet.

[51] Sak ME, Alanbay I, Rodriguez A, Gokaslan T, Borahay M, Shureiqi I (2016). The role of 15-lipoxygenase-1 expression and its potential role in the pathogenesis of endometrial hyperplasia and endometrial adenocarcinomas. Eur J Gynaecol Oncol.

[52] Kandoth C, Schultz N, Cherniack AD, Akbani R, Liu Y, Shen H (2013). Integrated genomic characterization of endometrial carcinoma. Nature..

[53] Zhang X, Chen D, Zhao X, Wang C, He Y, Chen Y (2023). Application of molecular classification to guiding fertility-sparing therapy for patients with endometrial cancer or endometrial intraepithelial neoplasia. Pathol Res Pract.

[54] Stelloo E, Nout RA, Osse EM, Jürgenliemk-Schulz IJ, Jobsen JJ, Lutgens LC (2016). Improved risk assessment by integrating molecular and clinicopathological factors in early-stage endometrial cancer-combined analysis of the PORTEC cohorts. Clin Cancer Res.

