# Supplementary material for: Genome-wide CRISPR screening reveals ADCK3 as a key regulator in sensitizing endometrial carcinoma cells to MPA therapy
Source: Br J Cancer. 2023 Jul 4;129(4):601–11. doi: 10.1038/s41416-023-02347-2 (PMC10421920; doi:10.1038/s41416-023-02347-2)
Supplement: Supplementary file 1 — Supplementary Figures and Tables [file 41416_2023_2347_MOESM1_ESM.pdf]

Supplementary Information

Genome-wide CRISPR screening reveals ADCK3 as a key regulator in sensitizing endometrial carcinoma cells to MPA therapy

Zhang et al.

This file contains:

1. 7 Supplementary Figures
2. 3 Supplementary Tables

Supplementary Figures

Fig. S1

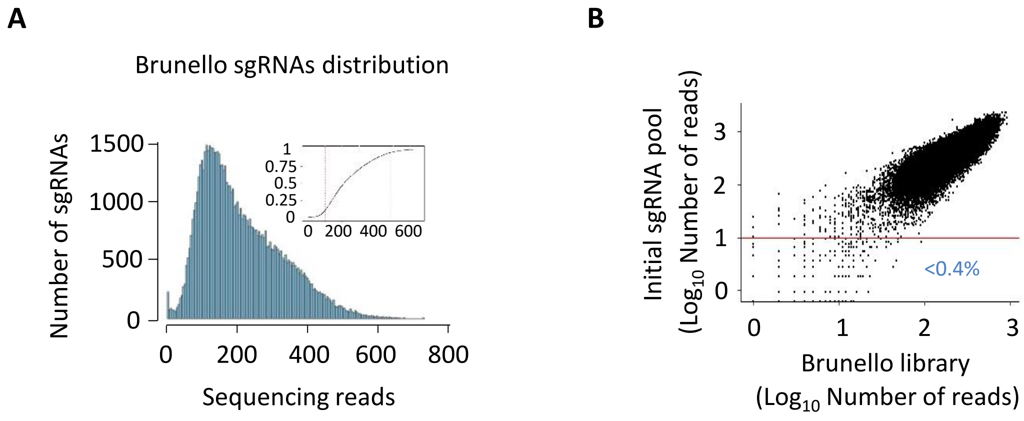

Fig. S1 Representative validation of the sgRNA library. (**A**) Histograms of sgRNA representation after electroporation. Inset: Cumulative distribution of sequencing reads. The number of sequencing reads for the 10th and 90th sgRNA percentiles is indicated by the dashed red lines. (**B**) Distributive correlation of the Brunello library and the sgRNA pool collected from the infected cells after puromycin selection (Initial sgRNA pool). The red line indicates that less than 0.4% of sgRNAs have undetectable representation (less than 10 reads).

Fig. S2

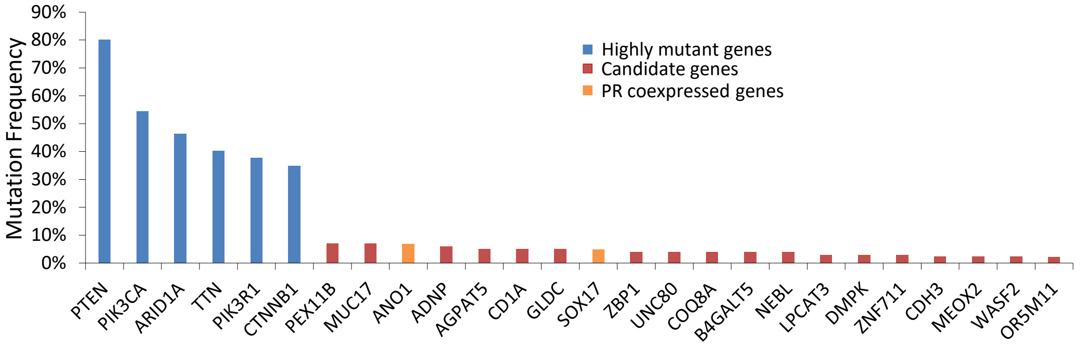

Fig. S2 The mutation of candidate genes in EC. The top 6 frequently mutated genes in EC are shown in blue bar; the top 18 candidate genes identified by CRISPR screening are shown in red bar; the PR (progesterone receptor) coexpressed genes are shown in orange bar.

Fig. S3

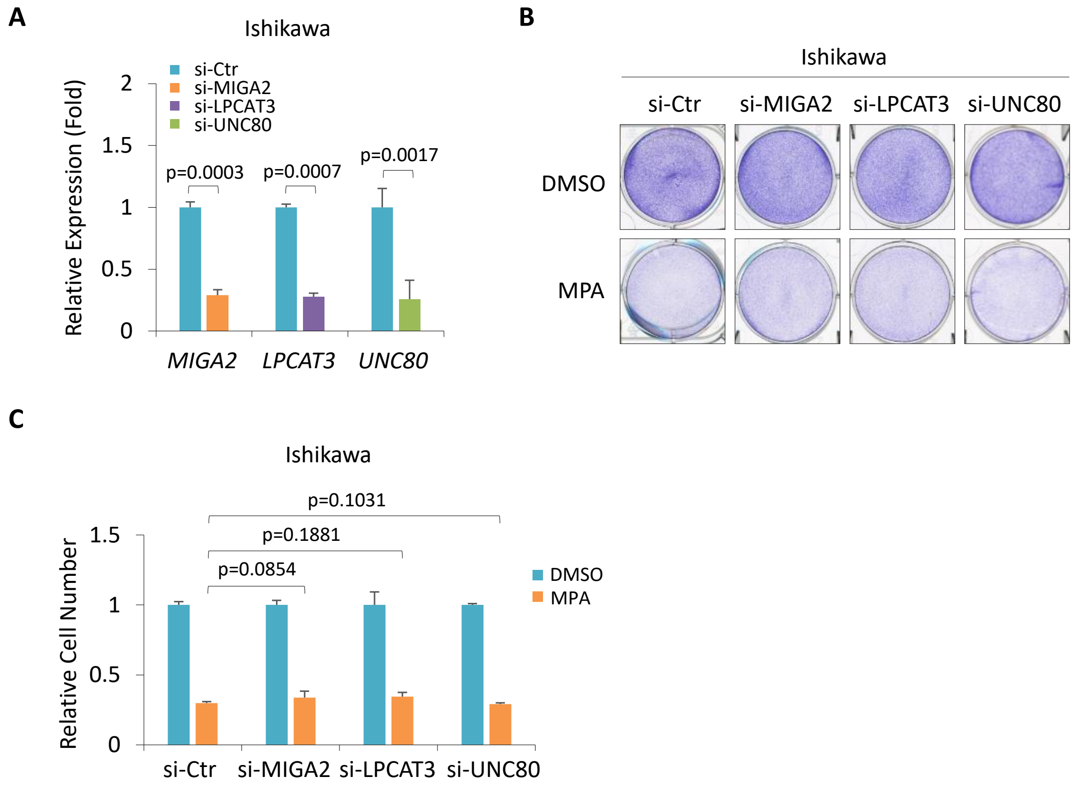

Fig. S3 MIGA2, LPCAT3 or UNC80 is not required for MPA-induced EC cell death. (**A**) RT-qPCR analysis of *MIGA2*, *LPCAT3* or *UNC80* expression in Ishikawa cells transfected with control siRNA or siRNA targeting MIGA2, LPCAT3 or UNC80 for 96 h. (**B**) Cell growth assay by crystal violet staining of Ishikawa cells with or without MIGA2-KD, LPCAT3-KD and UNC80-KD in response to 40 μM MPA treatment for 3 days. (**C**) Quantitative analysis of the cell number in (**B**). Data are shown as the mean ± SD, n=3 in **C**; n=2 in **A**.

Fig. S4

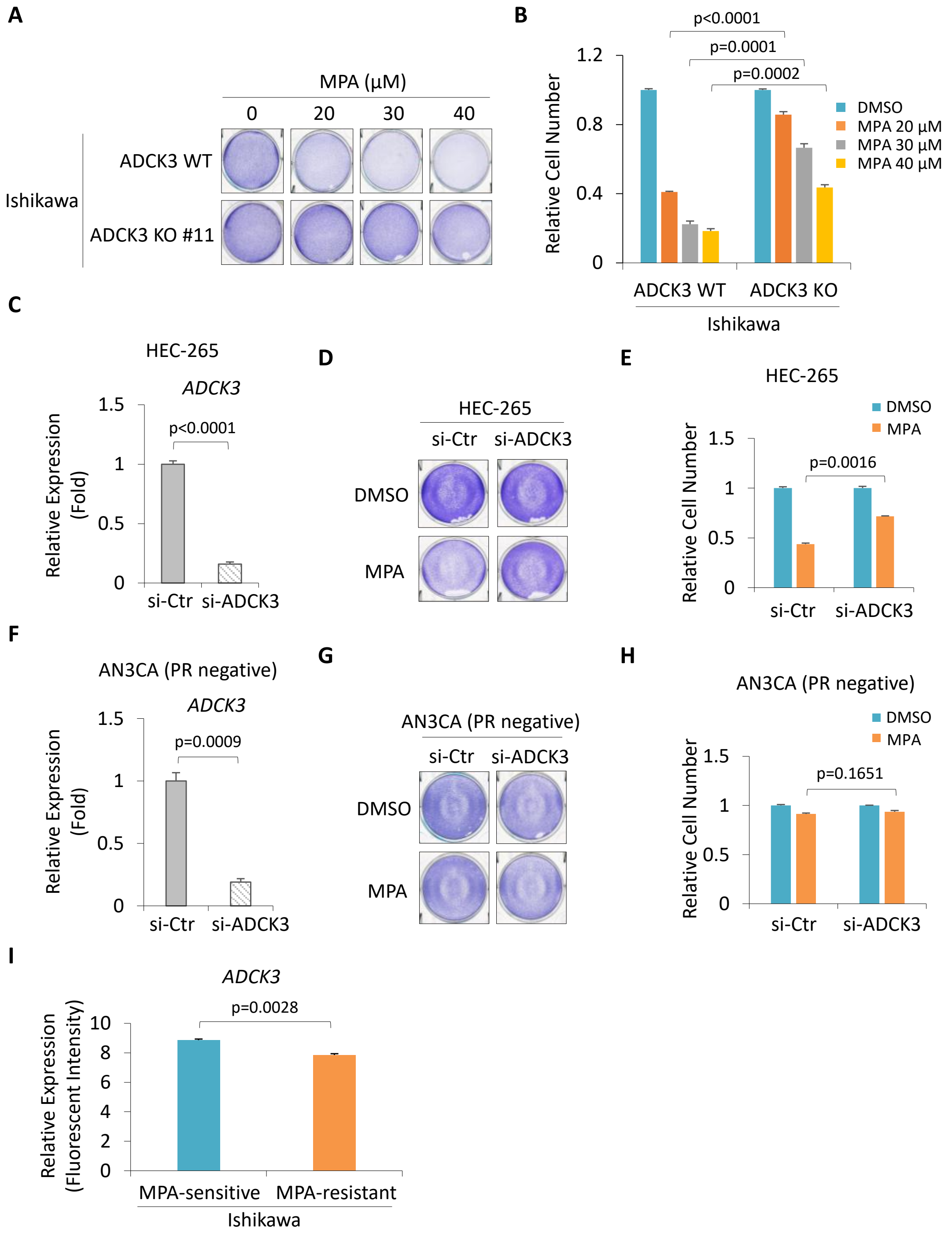

Fig. S4 Functional validation of ADCK3 in MPA resistance of EC cells. (**A**) Cell growth assay by crystal violet staining of parental or ADCK3-KO Ishikawa cells treated with or without increasing concentrations of MPA, as indicated, for 3 days. (**B**) Quantitative analysis of the cell number in (**A**). (**C**) RT-qPCR analysis of *ADCK3*

expression in HEC-265 cells transfected with control siRNA or siRNA targeting ADCK3. (**D**) Cell growth assay by crystal violet staining of HEC-265 cells with or without ADCK3-KD in response to 40 μM MPA treatment for 3 days. (**E**) Quantitative analysis of the cell number in (**D**). (**F**) RT-qPCR analysis of *ADCK3* expression in AN3CA cells transfected with control siRNA or siRNA targeting ADCK3. (**G**) Cell growth assay by crystal violet staining of AN3CA cells with or without ADCK3-KD in response to 40 μM MPA treatment for 3 days. (**H**) Quantitative analysis of the cell number in (**G**). (**I**) The expression of *ADCK3* in parental Ishikawa cells (MPA-sensitive) or in acquired MPA-resistant Ishikawa cells, analyzed based on GEO dataset (GSE121367). Data are shown as the mean ± SD, n=3 in **B**, **E**, **H** and **I**; n=2 in **C** and **F**.

Fig. S5

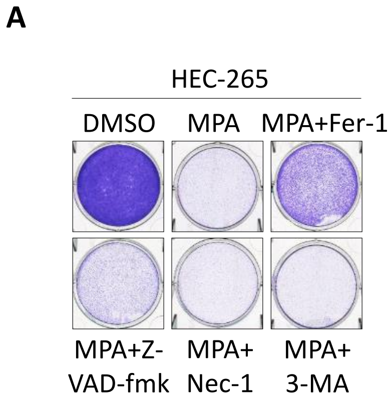

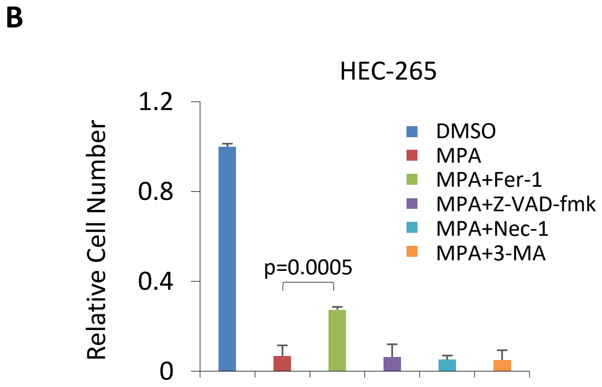

Fig. S5 MPA induces ferroptosis in HEC-265 cells. (**A**) Cell growth assay by crystal violet staining of HEC-265 cells treated with 40 μM MPA for 3 days in the presence or absence of Fer-1 (2 μM), Z-VAD-fmk (10 μg/ml), Nec-1 (10 μg/ml), or 3-MA (2 mM). (**B**) Quantitative analysis of the cell number in (**A**). Data are shown as the mean ± SD, n=2.

Fig. S6

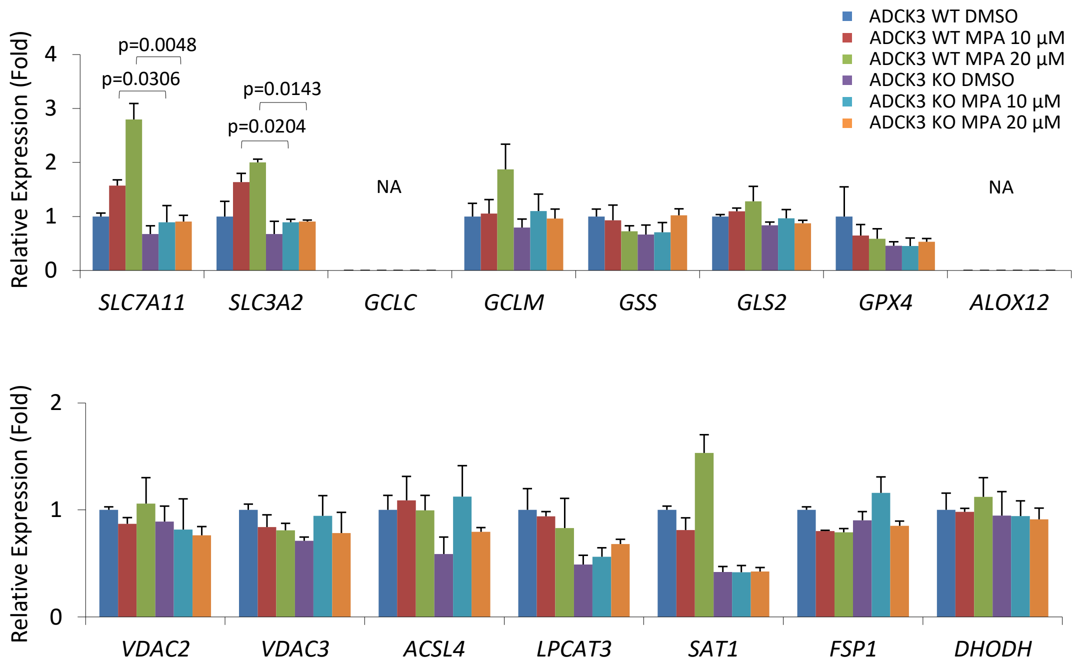

Fig. S6 Evaluation of ferroptosis-related genes in Ishikawa cells upon ADCK3 KO in response to MPA. RT-qPCR analysis of ferroptosis-related genes in parental or ADCK3-KO Ishikawa cells treated with increasing concentrations of MPA, as indicated. Data are shown as the mean ± SD, n=2. NA: the expression of indicated gene was not detectable.

Fig. S7

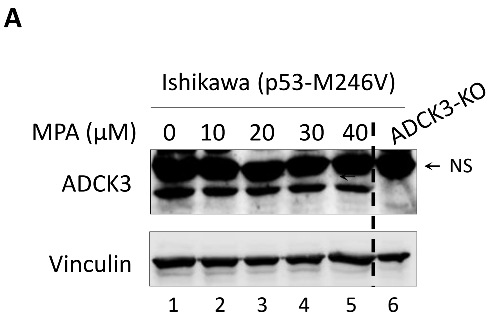

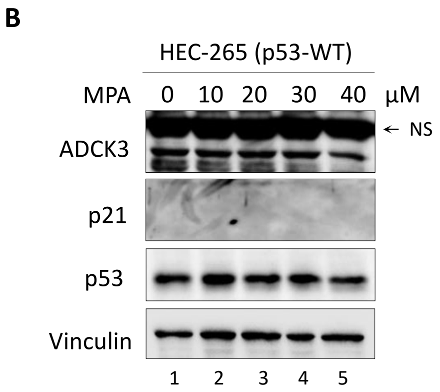

Fig. S7 MPA does not activate the p53-ADCK3 axis. (**A**) WB analysis of ADCK3 in Ishikawa cells treated with increasing amounts of MPA, as indicated, for 24 h. Western blotting of ADCK3-KO cell lysates served as a negative control. (**B**) WB analysis of ADCK3, p21 and p53 in HEC-265 cells in response to increasing amounts of MPA, as indicated, for 24 h.

Supplementary Tables

Tab. S1 The antibodies, siRNA, sgRNA and primers used in this study.

**Antibody**		
**Name**	**Catalog**	**Application**
ADCK3	Proteintech: 15528-1-AP	WB
Vinculin	Sigma: V9131	WB
p53	Santa Cruz: sc-126	WB, ChIP
p21	Santa Cruz: sc-6246	WB
**siRNA**		
**Name**	**Sequence**	**Application**
Control siRNA (si-Ctr)	UUCUCCGAACGUGUCACGU	Negative control of knockdown
Homo si-ADCK3	#1: GGAUAAACAUGAAGAAUAUUU<br>#2: GAAUCAACAACUUGUUUCAAU<br>#3: GCAUCAACAGUGAUGUCAA	Knockdown
Homo si-LPCAT3	#1: GGAAAGAGAAGUUAAAGAAGA<br>#2: GCUUCAAUGGCUUUGAAGAAA<br>#3 GCAAGUUUGUGCUGUACAA	Knockdown
Homo si-UNC80	#1: GAGAACAACCGAAGAAAGAUC<br>#2: GCAAAUAGGCCUUUAACUUGA<br>#3 GGAUAGAAUGUUCAACAAA	Knockdown
Homo si-MIGA2	#1: GUCGAAUAAACAAUUGAGACG<br>#2: CCUUCAUUAGGACAAGAAACU<br>#3 GGAAGGAGUUUGCAGAGAA	Knockdown
**sgRNA**		
**Name**	**Targeting sequence**	**Application**
Homo sgADCK3	CCGAATGAAGGGCGGCCGCG	Knockout

Primer		
**Name**	**Sequence**	**Application**
Homo *ADCK3*	F: GGATGATGCCTTTATCAACCCC<br>R: CCGCTCCTCGAAGTATTCCAA	RT–qPCR
Homo *LPCAT3*	F: GGAGCTGAGCCTTAACAAGTT<br>R: CAAAGCAAAGGGGTAACCCAG	RT–qPCR
Homo *UNC80*	F: TGTCCTTTGAGCGAGTGTTGG<br>R: TGTTTCGGTTTGAAAGCAGGG	RT–qPCR
Homo *MIGA2*	F: GAGGGCACGTCTATGATCCAG<br>R: CGGGACCAACCTGCTTCTTC	RT–qPCR
Homo *ALOX15*	F: GGGCAAGGAGACAGAACTCAA<br>R: CAGCGGTAACAAGGGAACCT	RT–qPCR
Homo *SAT1*	F: ACCCGTGGATTGGCAAGTTAT<br>R: TGCAACCTGGCTTAGATTCTTC	RT–qPCR
Homo *GLS2*	F: GCCTGGGTGATTTGCTCTTTT<br>R: CCTTTAGTGCAGTGGTGAACTT	RT–qPCR
Homo *SLC7A11*	F: TCTCCAAAGGAGGTTACCTGC<br>R: AGACTCCCCTCAGTAAAGTGAC	RT–qPCR
Homo *GPX4*	F: GAGGCAAGACCGAAGTAAACTAC<br>R: CCGAACTGGTTACACGGGAA	RT–qPCR
Homo *GCLM*	F: TGTCTTGGAATGCACTGTATCTC<br>R: CCCAGTAAGGCTGTAAATGCTC	RT–qPCR
Homo *GSS*	F: GGGAGCCTCTTGCAGGATAAA<br>R: GAATGGGGCATAGCTCACCAC	RT–qPCR
Homo *GCLC*	F: GGAGGAAACCAAGCGCCAT<br>R: CTTGACGGCGTGGTAGATGT	RT–qPCR
Homo *ACSL4*	F: CATCCCTGGAGCAGATACTCT<br>R: TCACTTAGGATTTCCCTGGTCC	RT–qPCR
Homo *SLC3A2*	F: TGAATGAGTTAGAGCCCGAGA<br>R: GTCTTCCGCCACCTTGATCTT	RT–qPCR
Homo *VDAC2*	F: GGCGTGGAATTTTCAACGTCC<br>R: AGACCATACTCACACCACTTGTA	RT–qPCR
Homo *VDAC3*	F: TTGTACCGAACACAGGAAAGAAG	RT–qPCR

	R: CCCAGCCATAGATGGTTGGTC	
Homo *ALOX12*	F: ACCAGTTCCTCAATGGTGCC<br>R: TCCTCGGATCACGTTGGCT	RT–qPCR
Homo *FSP1*	F: GATGAGCAACTTGGACAGCAA<br>R: CTGGGCTGCTTATCTGGGAAG	RT–qPCR
Homo *DHODH*	F: CCACGGGAGATGAGCGTTTC<br>R: CAGGGAGGTGAAGCGAACA	RT–qPCR
Homo *β-Actin*	F: GGCCAACCGCGAGAAGAT<br>R: GCCAGAGGCGTACAGGGATA	RT–qPCR
Homo *B2M*	F: GAGGCTATCCAGCGTACTCCA<br>R: CGGCAGGCATACTCATCTTTT	RT–qPCR
Homo *ADCK3*	F: TTTGGTCGCCAATACAGCTT<br>R: GAGCCTAGAGCCCCAGACA	ChIP–qPCR
Homo *p21*	F: AGCAGGCTGTGGCTCTGATT<br>R: CAAAATAGCCACCAGCCTCTTCT	ChIP–qPCR

Tab. S2 The list of candidate genes identified by CRISPR screening.

Gene	log2 Fold change	FDR
ADCK3	6.0548	0.00165
FAM73B	4.0897	0.00165
LPCAT3	3.6425	0.018564
B4GALT5	3.6018	0.058416
C21orf140	2.8743	0.607388
UNC80	2.6819	0.638614
CT47A10	2.4603	0.607388
MORN1	2.3461	0.912561
CDH3	2.2778	0.43255
GLDC	2.1431	0.743967
CPOX	2.1314	0.43255
OR5M11	2.1252	0.283003
SERTM1	2.0724	0.743967
ZBP1	2.0246	0.816154
DMPK	2.0058	0.912561
PEX11B	2.0008	0.816154
CD1A	1.9977	0.94761
C22orf24	1.9772	0.607388
SAG	1.9689	0.816154
AGPAT5	1.9475	0.816154
UQCR11	1.8929	0.816154
MEOX2	1.8847	0.816154
ETS2	1.8369	0.607388
CXorf65	1.8252	0.743967
EDIL3	1.7903	0.912561
TTC19	1.7859	0.874222
ZNF711	1.7792	0.816154
C10orf113	1.7549	0.743967
CLYBL	1.7379	0.842123
WASF2	1.7107	0.743967
ADNP	1.7069	0.816154
CLPP	1.6943	0.842123
SGK223	1.6303	0.882069
RILPL1	1.6133	0.816154
C5orf47	1.6034	0.838002
FKBP4	1.6022	0.816154
PRSS1	1.589	0.816154
MCCC1	1.5823	0.743967
C8A	1.5633	0.94761
PAK6	1.5528	0.94761
CHPT1	1.5519	0.912561

SOCS3	1.548	0.912561
ZNF768	1.5417	0.948005
CXorf21	1.5386	0.743967
CDHR5	1.5231	0.743967
OR8B3	1.5102	0.912561
COX5A	1.4889	0.816154
OR10K1	1.4837	0.841648
PPP6R3	1.4776	0.948005
CYP26A1	1.4674	0.842123
ZNF81	1.4665	0.948005
MAGEB1	1.4652	0.945825
ALAD	1.4637	0.94761
KRTAP15-1	1.4471	0.912561
ADRM1	1.4451	0.842123
ANKRD52	1.4425	0.912561
NAPB	1.4416	0.912561
KLHL18	1.4398	0.816154
STIP1	1.4347	0.912561
IAH1	1.4318	0.912561
MRGPRX2	1.4292	0.912561
CYLD	1.4155	0.94761
ZNF549	1.4019	0.912561
THSD7A	1.3993	0.638614
POR	1.396	0.94761
LAMP5	1.3959	0.816154
PAFAH2	1.3824	0.912561
C8orf4	1.3676	0.948005
C9orf163	1.3661	0.94761
ARVCF	1.3655	0.948005
PHKG1	1.3645	0.94761
POU6F2	1.3592	0.743967
APOL5	1.359	0.912561
SVEP1	1.3462	0.912561
OR10H4	1.3446	0.94761
TAS2R4	1.3439	0.912561
PEX14	1.3432	0.94761
LOC388813	1.3376	0.98216
PRIMA1	1.3254	0.912561
PGBD1	1.3205	0.912561
BHLHE41	1.3173	0.962122
UPB1	1.3141	0.816154
RDX	1.3122	0.912561
TTC23	1.307	0.842123
ATP8A1	1.3055	0.85045

TIMM44	1.2777	0.9934
OR5L2	1.2765	0.912561
C10orf82	1.276	0.962122
CCDC153	1.2675	0.912561
KIAA1024	1.2646	0.94761
TUBAL3	1.2603	0.994926
FGR	1.2602	0.888232
FAM109B	1.2571	0.9934
OCEL1	1.2469	0.948005
PROSER2	1.2448	0.842123
CT45A1	1.2307	0.94761
FBXO31	1.2291	0.912561
AGBL1	1.224	0.912561
FOXE3	1.2142	0.94761
PRRG3	1.2095	0.964339
ZDHHC5	1.2073	0.912561
CXXC1	1.2065	0.948005
OR7G2	1.2002	0.966345
UBE2O	1.1991	0.9934
ANKRD30B	1.1988	0.912561
OR6C74	1.1922	0.912561
OCSTAMP	1.1895	0.743967
NEUROD6	1.1876	0.842123
RAD18	1.1839	0.9934
GRM7	1.1803	0.743967
SULT1A1	1.1761	0.948005
NRG3	1.1735	0.948005
RASEF	1.1727	0.9934
SMAD7	1.1709	0.912561
AXIN2	1.1701	0.948005
SRRM4	1.1619	0.948005
GPC3	1.1605	0.948005
TWIST1	1.1582	0.967355
BTN3A1	1.1565	0.984037
UHRF1BP1L	1.1492	0.94761
TRPM6	1.1478	0.842123
CTBP1	1.1418	0.882069
FAM189A1	1.1317	0.94761
ADCK5	1.1262	0.816154
XCL2	1.1258	0.912561
OR51A7	1.1258	0.94761
PEBP1	1.1222	0.94761
KNDC1	1.12	0.948005
SEMA4G	1.1197	0.94761

ZNF550	1.1139	0.94761
EHD2	1.1138	0.948005
HIST1H2BH	1.1028	0.912561
ABCC1	1.0895	0.964339
MAGEA10	1.0872	0.912561
MSI2	1.0841	0.967355
UBQLNL	1.0813	0.816154
HOXA9	1.0776	0.94761
JARID2	1.0654	0.842123
SYT10	1.065	0.948005
TSPY2	1.0646	0.842123
C9	1.0626	0.951436
BMP5	1.0596	0.912561
PTCHD3	1.0593	0.94761
PRELP	1.0581	0.98216
OR10H5	1.0547	0.912561
SNAP25	1.0537	0.88783
DEK	1.0532	0.948005
SH2B1	1.0455	0.912561
IGFL2	1.0435	0.912561
SLC9B2	1.041	0.981634
CDKL5	1.0389	0.94761
USP25	1.0366	0.94761
FAM26E	1.036	0.912561
LRRC39	1.0336	0.948005
DUSP15	1.0268	0.912561
SHOX	1.0263	0.979464
MMEL1	1.0256	0.9934
LMNA	1.0246	0.94761
SLC10A6	1.023	0.948005
PTRHD1	1.0223	0.985177
TAS2R3	1.0129	0.912561
DMRTC2	1.0119	0.912561
LPCAT1	1.0098	0.994156
NUDT3	1.0047	0.948005
ZCCHC3	1.004	0.948005
KRTAP21-3	1.0024	0.924028

Tab. S3 The list of candidate gene-associated sgRNAs identified by CRISPR screening.

sgRNA	Gene	log2 Fold change	FDR
ADCK3_44814_TGTCCGAGGCCAATGCAGAG	ADCK3	6.8164	0
C21orf140_76363_CATTATGTGTTGCCCAGAGG	C21orf140	6.7148	0
THSD7A_65937_CTGCGAGCCAGATAACGGAA	THSD7A	6.6859	0
ADCK3_44811_CCGAATGAAGGGCGGCCGCG	ADCK3	6.5038	0
UPB1_39394_CTGTACATAAGCCAGTTGAG	UPB1	5.8358	0
UNC80_68047_GGGAATGAGTAATGCCGGCG	UNC80	6.1356	0
ADCK3_44813_CCTGGGTCATGCCCACAGCG	ADCK3	5.6707	0
FAM73B_54402_GGTGACAGGCCTGATGACCA	FAM73B	5.4124	0
PEX11B_23016_TCTCCACAAGTTCTACGCCT	PEX11B	6.3911	0
TRPM6_60983_GGATGGTAAGAAAGCGATGG	TRPM6	5.3512	0
ADCK3_44812_CCTGGCTAAGATCTTCGAGC	ADCK3	5.2282	0
SH2B1_34222_GGTCCTCAGATGATCGAGAA	SH2B1	6.1561	0
B4GALT5_24524_TTCGGAGTGCTTATGCCAAG	B4GALT5	5.4234	0
FBXO31_49953_CCAGATATCGGGCCATACGG	FBXO31	5.6604	0
DMPK_4866_CGGACGCGGGGCGTTCAGCG	DMPK	5.5898	0
B4GALT5_24523_TACTTCGTCTATGTGGCGCC	B4GALT5	5.1246	0
IGFL2_61894_CTGAGCGAGACCCGCCAATG	IGFL2	5.1821	0
EDIL3_26954_GTTACTGGTGTGATTACCCA	EDIL3	5.2151	0
ANKRD52_67331_GGCCATCATGAATGGCCATG	ANKRD52	5.1122	0

LPCAT3_27214_GCGCGTCAGAACAGGCGCTG	LPCAT3	4.8013	0
PRIMA1_61401_ACCACCAGCTTTCCTCAGTG	PRIMA1	4.3773	0
FAM73B_54401_GCAGAGAGCCTGTACATGCA	FAM73B	4.3642	0
OR8B3_71748_TGAGCATAGAACAGACCTGA	OR8B3	4.7899	0
WASF2_27220_TCGGTCGACCCTCTCAGCAA	WASF2	4.7567	0
MORN1_50329_GTCATGGAGTACAAAGCCGG	MORN1	5.0485	0
PROSER2_66469_CACCCCCGATCCCCCCAGGA	PROSER2	4.7071	0
FAM73B_54400_GACAGCATGCTGCTAGACCT	FAM73B	4.3076	1.24405729622826e-320
ZBP1_51484_CACCTGGTGCCATTGAAGGG	ZBP1	4.5693	3.15029999998014e-313
UQCR11_29732_GAGCTGGTCAAGAACTGGTG	UQCR11	4.1566	2.73E-252
LPCAT3_27216_TGGTGCGGCCCATTAGTCGA	LPCAT3	4.3858	1.01E-247
NAPB_47117_CTCACCCAAACAGCCCTCGG	NAPB	4.2057	2.28E-227
SAG_16793_GGCAAGTAGTCAGGAAACTG	SAG	4.4207	3.93E-215
CYLD_4332_TGAGACTGAATGGTAAAGAG	CYLD	4.5333	9.74E-200
USP25_36579_ACAAAATCTCTCAAATCCGG	USP25	4.3657	5.32E-199
CD1A_2531_CCAGGGGCACAGGAAAACGA	CD1A	4.7886	1.70E-194
OR51A7_58170_ATACATGGGCTCATGAAGCG	OR51A7	4.5262	9.32E-191
SULT1A1_18471_GGGGCTTTGAACTCAAGGAA	SULT1A1	4.1099	7.79E-166
OR5M11_65294_GAAGCCAGCAGATATGAACA	OR5M11	3.8462	9.24E-126
LRRC39_59362_CCTGGTTTCCTAGGCTAGTG	LRRC39	3.9186	8.32E-111
NRG3_28974_CGCTACAGCGTAACTCCCGG	NRG3	4.2672	3.03E-110
CDHR5_39670_TAAGACCAAGGAGATAAGGG	CDHR5	4.4206	8.08E-105

BHLHE41_49184_GACTTGGATGCGTTCCACTC	BHLHE41	3.6784	3.36E-97
ARVCF_1229_AAGATGGTCATCATTGACCA	ARVCF	3.5841	3.00E-94
C10orf82_61167_GCGCTATAAAGAACAGCTGC	C10orf82	3.6166	5.26E-90
CT45A1_73500_CTTACTCTTTGCTTTGGACA	CT45A1	3.81	2.21E-85
GLDC_7205_AAACCTGTTGAACACTTGAT	GLDC	3.7814	5.38E-82
MSI2_58766_ACCTTGGGTTGCGCTCGACG	MSI2	3.5386	1.42E-80
OCEL1_49439_ACAGCTCATAGTCGGGCACT	OCEL1	3.8397	1.23E-75
TWIST1_19758_CCCCCGCGCTTGCCGCTCGG	TWIST1	3.6927	2.55E-75
PAK6_44596_GCACCCGTGTGATTCGCGAA	PAK6	3.7602	3.96E-75
CLPP_21381_AGAGGAGCTGTGCGATAACA	CLPP	3.6502	1.83E-73
WASF2_27219_GGGCCTGGTGGAGAGCCTAG	WASF2	3.7063	4.10E-73
THSD7A_65938_GCCAGAGCCTCCGAACTGCG	THSD7A	3.7101	1.61E-71
OR10H4_59177_AAAGGTGATGGAATGATGGG	OR10H4	4.02	7.47E-71
C8orf4_44479_ACGTGGACATGATGACGGCT	C8orf4	3.4762	7.13E-70
GPC3_7190_CTGTGGCGGTTACTGCAATG	GPC3	3.5329	6.72E-69
LPCAT3_27213_CTGTTGACTACTTTGACGGA	LPCAT3	3.4037	2.11E-67
MEOX2_11370_GCGGAGGCGGAGAAGCGAAG	MEOX2	3.8628	7.40E-67
CT47A10_74531_CCTCTGGGGCAGCAGCAGCG	CT47A10	3.5208	1.35E-64
CCDC153_67225_GAAGGGAAGGCCATATATGC	CCDC153	3.4649	2.95E-62
ZNF81_69803_GTTCGAAGTTCCTAAACCAG	ZNF81	3.49	2.56E-60
CDHR5_39669_GTTGGTAATTCGATATGTGA	CDHR5	3.4982	9.45E-60
CHPT1_44802_GCTGGGGCTCGCCGTCAACG	CHPT1	3.434	1.18E-58

C8A_2022_ATCTGGTTTAGTACCGACAC	C8A	3.2942	1.91E-56
LOC388813_71199_GTTCACTCTCTCCAAAGGGT	LOC388813	3.4287	6.30E-52
PRELP_14727_GAATCCGCAAGATAGACCAG	PRELP	3.5395	7.94E-52
TAS2R4_37370_TAGTCCCAGCATAAGAAACC	TAS2R4	3.5031	4.11E-50
ZNF768_49754_GGAGCCCCGGGTATGAACCC	ZNF768	3.6037	8.54E-48
IAH1_68039_TCCCCGAAGAGCAACAAGCG	IAH1	3.2737	1.82E-47
PEBP1_13228_GCATGTCACCTACGCCGGGG	PEBP1	3.4282	3.22E-45
ADNP_32677_TGGAGACTGATTAAGCCGAG	ADNP	3.3965	3.64E-45
MRGPRX2_57930_GTTTCCTACCAGCCCGACCA	MRGPRX2	3.3034	1.58E-44
PEX11B_23014_CGAAGTACAAGGCTCGATTG	PEX11B	3.2431	2.65E-42
OR10H5_67865_ACTTCAACAGAGGTGGCACG	OR10H5	3.3796	6.30E-42
PEX14_13700_TATGGCTGAGATATGAGGTG	PEX14	3.1779	7.71E-42
ZDHHC5_34089_CTGTCCCCACGACTCAACTG	ZDHHC5	3.0596	2.30E-41
C21orf140_76362_ATGCACAATACACCAAGTTG	C21orf140	3.3291	2.18E-40
PRSS1_15038_GAGTCTTCCTGTCGTATTGG	PRSS1	3.3643	5.45E-39
C5orf47_60161_CCTCCACACCAGGGGTCGTG	C5orf47	3.0362	8.05E-39
PTCHD3_70249_AGGAGCATTACACCCCTGTG	PTCHD3	3.1245	8.29E-39
CT47A10_74530_CCGAGGTGGTCGGAGTCGCA	CT47A10	3.1954	1.73E-38
RASEF_63128_GCCACGCGCGAACTCCTGGA	RASEF	3.0322	3.92E-38
AGPAT5_42332_CTTCTTCGAGAATTACACCG	AGPAT5	3.3458	4.48E-38
BTN3A1_30172_ATACAGTGGAGCAACAACAA	BTN3A1	3.1806	1.99E-37
AGPAT5_42334_GCAAAGTAACACCCATACAA	AGPAT5	3.2325	2.85E-37

UQCR11_29731_CAGGATCAGCCGCCAATCGG	UQCR11	2.8921	3.18E-37
SAG_16794_TGATTATTACGTCAAGCCCG	SAG	3.3363	4.62E-37
MCCC1_44587_AAGGTCCTCATTGCAAACAG	MCCC1	3.2922	5.85E-36
LMNA_10707_GCTGACCACCTCTTCAGACT	LMNA	3.6205	8.89E-36
OR5M11_65296_TGGATCTACAGAAGGTCAGG	OR5M11	3.1384	1.11E-35
KRTAP15-1_66531_CTGACAGCCATTGTAGAGAG	KRTAP15-1	3.0625	2.60E-35
CPOX_3815_GATTCAAGTATGTTGGAGTG	CPOX	3.3665	3.37E-35
STIP1_29693_GTGAGAAGGCCATTGAAGTG	STIP1	2.8845	4.50E-35
ZNF550_63585_AAGACGCCTTGATTCATGCA	ZNF550	2.828	3.69E-34
EHD2_37142_TGGAACATCACATCTCCCCT	EHD2	2.9937	8.35E-34
UBE2O_47078_GAGCCTCAAGAACATGACTG	UBE2O	3.321	1.13E-33
ZNF549_66699_GCAGAAACCTTATACGTCTG	ZNF549	3.1334	1.34E-33
SLC9B2_60143_TGAACTGATTAGGTTACCTG	SLC9B2	3.075	3.06E-33
CLYBL_64286_ATCACAAGTACATCCCCCGG	CLYBL	3.3543	4.98E-33
AXIN2_21568_TTACTGCCCACACGATAAGG	AXIN2	2.9645	8.54E-33
KNDC1_54932_CGAGTCCACATCCGACACCG	KNDC1	3.2541	1.48E-32
DMRTC2_47205_GGCACCGGAGTCCAGGACAA	DMRTC2	3.117	9.53E-32
TRPM6_60984_TCTGGAATAGGCTCAAGACA	TRPM6	2.9207	1.59E-30
CDH3_2839_TGGTGAACATGAGGTCGTGT	CDH3	3.0097	7.95E-30
C9_2039_GAACTCACTACAGTAGCTCT	C9	3.0773	1.85E-29
LAMP5_33610_TTATTGTACCTTATGATGTG	LAMP5	2.9661	5.22E-28
PPP6R3_42224_GGAGTCTTGATCGGTAATTG	PPP6R3	3.371	1.28E-25
C22orf24_33676_GTTGTCATGACATTGT	C22orf24	2.8744	2.03E-24

CTGC			
UHRF1BP1L_31648_CTCTGAATAGAAATTGCACG	UHRF1BP1L	3.0718	8.10E-24
RAD18_44445_TAGGATGCAAAGCATCGCAT	RAD18	2.764	4.77E-22
SRRM4_53760_CAAACTGGACTTCTTCACTT	SRRM4	2.7435	2.42E-21
OR5M11_65293_GAACAAGAAAGCTTAATGAG	OR5M11	2.9747	4.25E-21
FAM109B_62326_GCTCGGTGGTTAGGAGCCTG	FAM109B	2.7366	7.69E-21
XCL2_18556_TGCTGATCCACAAGCCACGT	XCL2	2.9273	3.30E-20
KLHL18_32317_TCACCACAGCCACGCCAACG	KLHL18	2.7283	1.50E-19
CXorf65_63234_TACGTTTGTAAGGTGGAACG	CXorf65	2.6123	3.24E-19
TTC19_40950_GCCAACTTAGCATTTATACG	TTC19	2.9966	6.89E-19
BMP5_1786_AGCTGAATTCCGGATATACA	BMP5	2.7286	7.53E-19
SEMA4G_46311_CTACCCACCTCTATGCATGT	SEMA4G	2.6799	1.45E-18
LPCAT1_50268_GAAGAGCGTCAGTGTCATGA	LPCAT1	2.8836	1.70E-18
PAFAH2_13257_CAACATTGACATGAGCCGTG	PAFAH2	2.6162	1.74E-18
RDX_15976_CTCGTCTGAGAATCAATAAG	RDX	2.8106	2.78E-18
RILPL1_70051_CGTGGCCGAGCTGACCGTCA	RILPL1	2.7034	8.14E-18
UNC80_68048_GTGCCGAGAAGTTAGCACCA	UNC80	2.9337	1.22E-17
SOCS3_23608_TTGAGCACGCAGTCGAAGCG	SOCS3	2.8999	5.74E-17
CXXC1_37105_ATGCGCCCTAACTTCTGTGA	CXXC1	2.5164	9.55E-17
B4GALT5_24522_CTTCTGATTGCATGCCTCGG	B4GALT5	2.7704	1.66E-16
SVEP1_50533_CAGAAATGAGCTATACAACG	SVEP1	2.6661	2.45E-16
CXorf65_63236_TCGATTTGTGTGAACAAACG	CXorf65	2.5887	2.90E-16
HIST1H2BH_21677_CTACTCCGTATACG	HIST1H2	2.6283	3.99E-16

TTTACA	BH		
SERTM1_72178_CACACTTCCCGAAAATCCTG	SERTM1	2.78	5.81E-16
ANKRD52_67330_GATGCCCACTATGCACTGCA	ANKRD52	2.6676	7.00E-16
PHKG1_13875_TCCATGAATGAGGACCACCC	PHKG1	2.4901	7.08E-16
SGK223_62998_GCAATGTTCCTCTCGCCGCG	SGK223	2.6403	7.96E-16
POR_14430_ACGGATTCTTGGCATCAAAG	POR	2.6724	1.20E-15
PROSER2_66471_GATGACATCTTCAGGCTCGG	PROSER2	2.7392	1.85E-15
TAS2R4_37367_ACAGTACAAGATATTGAGCA	TAS2R4	2.4696	2.79E-15
ZNF549_66700_GGGAAAAGATTGTCTGACAG	ZNF549	2.6522	3.68E-15
SERTM1_72179_GCAGAAACGCTAAAAGGCTG	SERTM1	2.7113	4.34E-15
C9orf163_63101_ATATCCGTGCCACGCCAGCA	C9orf163	2.2382	4.40E-15
EDIL3_26956_TGGGCAATGTACAAAGTGAA	EDIL3	2.5781	4.93E-15
C10orf113_70791_CTCTCTCTCATATAAGCTAG	C10orf113	2.6969	9.98E-15
CYP26A1_4449_ACAAGACGCATCTGTTCGGG	CYP26A1	2.6078	1.35E-14
KIAA1024_32233_GGACGACTGTATCACTCCCG	KIAA1024	2.9329	2.76E-14
ZCCHC3_54837_GGCCGTCGGAGAGATCCGGC	ZCCHC3	3.1429	3.54E-14
TTC19_40947_AGCAGCCGTCACAGGCACAA	TTC19	2.5326	3.44E-14
NAPB_47115_AAGATGGCTAAAAATTGGAG	NAPB	2.5503	8.69E-14
FAM189A1_32567_GATGAACTCGTCGAAATCCA	FAM189A1	2.4123	8.85E-14
KRTAP15-1_66530_ACAGGATGTCTGATAGGATC	KRTAP15-1	2.5194	1.03E-13
ZDHHC5_34090_GAACCACGTGAAATCCCGTG	ZDHHC5	2.5044	1.05E-13
HOXA9_8660_GTGGAACCCAGTGCACGCGG	HOXA9	2.3723	2.00E-13
GLDC_7208_TGGCAGCCATATTCGCCA	GLDC	2.777	3.17E-13

AG			
CHPT1_44801_GAGTCCAGGTGACAAG ACAC	CHPT1	2.5172	4.25E-13
MMEL1_49137_CGAGAGTACTACTTCA ACGG	MMEL1	2.6016	1.59E-12
CDKL5_18415_GACTAAGACTAGCTCC AGCA	CDKL5	2.2611	1.84E-12
SMAD7_10979_GTGCGTGGTGGCATAC TGGG	SMAD7	2.7399	2.07E-12
PRRG3_48824_GGGTACTGTGCATAGCC AAG	PRRG3	2.4841	3.10E-12
FAM73B_54399_CTGGCCAAGAGGATA GGGAG	FAM73B	2.2745	3.06E-12
TIMM44_28191_TATAAAAAAATTCCGT GACG	TIMM44	2.9141	4.30E-12
TUBAL3_50174_TTTCTCCAGGCAGAAC AGTG	TUBAL3	2.6255	5.48E-12
FOXE3_6401_CAAAGCGTTCGGTGATG AAG	FOXE3	2.3146	5.57E-12
DEK_21061_AAGTGTCCAATATAAAAA GA	DEK	2.7117	6.06E-12
ETS2_5843_GCTGTTAATCCAATGAGGA A	ETS2	2.5315	7.72E-12
MCCC1_44589_CGACCAGCTTCGCAAT CATG	MCCC1	2.5317	8.91E-12
RDX_15974_ATAAAAAAGGAACTGAAT TG	RDX	2.5899	1.06E-11
STIP1_29694_TAATGTAAGTCATGTTAG TG	STIP1	2.6025	1.17E-11
MORN1_50328_GCCCCCGGGTACCTTG TAGG	MORN1	2.5527	1.73E-11
XCL2_18555_TGAGAGAGCAGATGCCA AGG	XCL2	2.6674	2.26E-11
CT47A10_74532_GGCCGGAGCTGGTAA CCAGG	CT47A10	2.502	2.24E-11
SLC10A6_69675_CCCGTGACATGGCCA ATCAA	SLC10A6	2.3394	2.39E-11
SMAD7_10977_CGAATTATCTGGCCCCT GGG	SMAD7	2.6323	3.20E-11
UNC80_68045_ACTTTGGCGTCATTCAA CGT	UNC80	2.6538	1.19E-10
ALAD_578_ATGGATTGCCTCAATAGCT G	ALAD	2.397	1.36E-10
SHOX_17328_GGTCCTTGAACAAATGC	SHOX	2.4922	1.69E-10

ACC			
PAFAH2_13260_GTATTTCAGGGACCGG TCAG	PAFAH2	2.2962	1.93E-10
NUDT3_30335_ACCCGCACCTACGACG GCGA	NUDT3	2.3603	1.97E-10
APOL5_51414_GTCAACTGTCCACAGTG ATG	APOL5	2.3021	3.60E-10
FKBP4_6363_AGGCTATGCTAAGCCCA ATG	FKBP4	2.4784	6.52E-10
OR10H4_59179_TAGACCCACAGAAAGT GAGG	OR10H4	2.3661	6.47E-10
BMP5_1788_CTAGTGGGTTATGCAAAA GG	BMP5	2.3359	1.17E-09
C5orf47_60164_TGCAGTGGCGTCCTGC AACT	C5orf47	2.0155	5.67E-09
COX5A_24657_ACCACCCGGGCCGACC CTCG	COX5A	2.1528	7.02E-09
ANKRD30B_70336_GAATTATGAGTGTT TACCTG	ANKRD3 0B	2.1617	9.32E-09
UHRF1BP1L_31650_TGCAGATTTCAAT ATATACC	UHRF1BP 1L	2.5383	1.17E-08
MCCC1_44590_GTCATGATTAAAGCCG TCCG	MCCC1	2.3758	1.52E-08
OR7G2_71863_CAGATTCATCATCAACA GCA	OR7G2	2.2943	1.90E-08
FGR_6319_CGAGTTGAACTGAACCCGT G	FGR	2.2499	2.10E-08
CDH3_2840_TGTGGGCACCCTCGACCG TG	CDH3	2.3182	3.08E-08
CXorf21_50977_GTAGAAATGGAATCCT CCAT	CXorf21	2.4638	4.16E-08
FOXE3_6402_CGAACATGTCTGCGGCC GCG	FOXE3	2.0066	5.46E-08
ZNF711_20464_TAATGGACACTTAGAA CATG	ZNF711	2.3253	5.84E-08
KIAA1024_32234_TTTCAATAGAAATCC CTCCG	KIAA102 4	2.485	6.25E-08
ADRM1_29952_CACGAACTCTCTGCGC TAGG	ADRM1	2.3829	6.28E-08
MAGEB1_11042_AGTATCCCCTAAAAC AGGAG	MAGEB1	2.4301	7.22E-08
CDKL5_18413_AAAGTCCGTGGACATG TGGT	CDKL5	2.0404	7.06E-08
PEBP1_13226_CCCGGATGCTCCCAGCA	PEBP1	2.6495	1.08E-07

GGA			
PHKG1_13874_TCAAGGTGACCTTCTCA GTG	PHKG1	2.3627	1.05E-07
SEMA4G_46312_GCTCCGGAATTCATAC CTAG	SEMA4G	2.2053	1.19E-07
CTBP1_4179_TGACAACATCGACATCA AGT	CTBP1	2.1598	1.33E-07
GLDC_7207_TCATTCCGAAATCAGCAC AT	GLDC	2.3085	1.84E-07
KLHL18_32318_TGGACTTATCTACGCT GTAG	KLHL18	2.1527	1.99E-07
ANKRD30B_70333_ACAGGCTCTACAAT GCGAGA	ANKRD3 0B	2.2107	2.61E-07
CDH3_2837_AAGACACAAGAGAGATTG GG	CDH3	2.2729	2.68E-07
CXorf65_63233_GCCAAGGCTCCGGATT CTTG	CXorf65	2.0606	4.44E-07
ZNF711_20463_GAGACTGATGTAGTAA CAGA	ZNF711	2.3519	6.74E-07
PEX14_13697_AGGGTGACGGTGACTTG ACT	PEX14	2.3796	7.80E-07
SERTM1_72180_TGGGTCCACTGACGTG GACA	SERTM1	2.2324	1.08E-06
OCEL1_49442_TCCGACAGCAGATCCAG GCT	OCEL1	2.2838	1.46E-06
OR10K1_71929_AGATGCCAGTTTAAGG ACAG	OR10K1	1.9868	2.29E-06
POR_14432_TCGTACAGCACGTTGGTAC G	POR	2.0082	2.47E-06
TAS2R3_37364_GCCAGGGACACAATTA AGGG	TAS2R3	2.1115	3.55E-06
ZCCHC3_54835_AAGAATCTAGCCGAG AAGAA	ZCCHC3	2.2652	3.90E-06
SH2B1_34219_ACTGACCAGTCCCCTAC CAA	SH2B1	2.2019	3.90E-06
ATP8A1_27947_GTTTAAGAAGTGCACC ATAG	ATP8A1	2.1235	5.04E-06
GPC3_7189_CATGTACAGAATCTATGA CA	GPC3	2.1661	5.10E-06
CPOX_3814_CCCCGCGCCTGGTCCCAG TG	CPOX	2.1934	6.88E-06
PPP6R3_42226_TCAAAGGGGAATCGTT TAGG	PPP6R3	2.0703	7.33E-06
LPCAT3_27215_TAGTGATTCATTGAGA	LPCAT3	1.9791	9.57E-06

ACTG			
CD1A_2530_AGGATGCGATCCAGGTGACA	CD1A	2.0772	1.20E-05
CYLD_4330_ATATTCAAGATCGTTCTGTG	CYLD	1.903	3.21E-05
CXXC1_37106_CCAAGCTAGAGATTCGCTAT	CXXC1	1.9048	3.49E-05
ARVCF_1231_CGGCCGCATTGGCCTTCACG	ARVCF	2.0697	3.98E-05
SVEP1_50534_GATGGGAAATTATCTGACGG	SVEP1	1.923	4.71E-05
PTRHD1_71981_ACAATACTTGGTGTTACGAA	PTRHD1	2.2403	5.54E-05
NRG3_28973_CCGGATCCCATAAACACTGT	NRG3	1.9461	5.95E-05
ABCC1_11741_AAAATGTGATTGGCCCCAAG	ABCC1	1.8559	6.10E-05
OR6C74_66502_CCCAGTGATGCTCAACATGT	OR6C74	1.8037	9.35E-05
CLYBL_64288_TTGTGCAGTGCTCGACTGTG	CLYBL	1.7536	0.00017734
NEUROD6_47239_AACGCCAGGAGTTTCCTGAT	NEUROD6	1.6606	0.00018112
OR5L2_34998_TTGCACATGTGGAGTCACTG	OR5L2	1.7137	0.00019792
KRTAP21-3_75667_TGTGGCTGTAACGGTTATTA	KRTAP21-3	1.6726	0.0002396
MAGEA10_11031_GGGCTCCAGCAGCCAAAAGG	MAGEA10	1.6951	0.00029305
IAH1_68037_AATCGCCTGAACTCTGTTGT	IAH1	1.7747	0.00029656
BHLHE41_49183_AGGATGAAGCTGGATTCCCG	BHLHE41	1.9077	0.00030354
SOCS3_23607_TCAGCGTCAAGACCCAGTCT	SOCS3	1.7823	0.00030666
AGBL1_58632_GTAGGTATAGGGATAGTGGT	AGBL1	1.91	0.0003501
C9orf163_63104_GAGTTAGGGAGCACGTGGTA	C9orf163	1.9204	0.00039703
PRRG3_48823_GGAATCGTTTCAGGACCGAA	PRRG3	1.8439	0.00042095
KLHL18_32316_TAATGCAAGGAATGGACCCA	KLHL18	1.6964	0.00044749
SYT10_69315_GGAGCACTTGAAATGCT	SYT10	1.8543	0.00048159

CTG			
C8A_2021_AGAATGAAGTGTCTTGTGAG	C8A	1.8659	0.00057547
PGBD1_53783_CCAGGGATGAGATGTGCATG	PGBD1	1.7945	0.00057799
OR5L2_34997_TGGTCACCATGTACAGCAGG	OR5L2	1.6193	0.00062404
OR7G2_71864_TGATGACTGTGTATCTAAGG	OR7G2	1.6761	0.00066401
ABCC1_11742_AACCTGACAGCATCGAGCGA	ABCC1	1.7319	0.0007232
OR8B3_71747_CTGAGTCATGCACCCAACAT	OR8B3	1.7257	0.00080738
PGBD1_53786_TTCTGCTACCAGGAGGCTCA	PGBD1	1.7004	0.0010522
SNAP25_17822_CTGCTCGTGTAGTGGACGAA	SNAP25	1.6656	0.0010504
TTC23_48267_AGGTGGAGTTTCTCTCGAGG	TTC23	1.8493	0.0012812
ALAD_577_AGACTCACGTGACAAAGATG	ALAD	1.9892	0.0013332
FAM26E_66436_TTCTATGAATGTGCCATGAG	FAM26E	1.7073	0.0013987
ETS2_5844_TTGCTGCACGGGGTTAACAA	ETS2	1.8094	0.0015208
C10orf113_70790_CGCAGCCACCTGGCAAGCGT	C10orf113	1.6536	0.0018849
ATP8A1_27945_AGTGTTGTATAACGACCTGT	ATP8A1	1.8295	0.0022067
AGBL1_58629_AGAGGAACTGATGCAATATG	AGBL1	1.7621	0.0023362
C22orf24_33677_TGGGAGGCTGCAATGATGAA	C22orf24	1.9335	0.0028964
SGK223_63000_TGGAGTTTGAATGGTCCGTG	SGK223	1.9777	0.0030621
PROSER2_66472_TGGCGCAGAAGATTTCCGAG	PROSER2	1.6947	0.0029119
RILPL1_70052_GAAAGCCCTGATCGAACAGA	RILPL1	1.6167	0.00345
DMRTC2_47204_CCAGCCTCAGACCCCCCATG	DMRTC2	1.714	0.0035074
CTBP1_4177_GATGTGGCACAGCGTCGAGT	CTBP1	1.7715	0.0035561
POU6F2_30696_CAACTAGTTACTAATG	POU6F2	1.8861	0.0038924

CACA			
OR6C74_66501_CATGATGGTGGTGTAATGCA	OR6C74	1.76	0.0042203
SYT10_69313_AAACAACAACCAGCATTGGG	SYT10	1.9076	0.0046962
ZBP1_51486_TGGGACACAGCAATGAGATG	ZBP1	1.7308	0.0047548
MEOX2_11372_TCCCGCGATTATGCAAGATG	MEOX2	1.8351	0.0049279
FAM189A1_32568_GCAGGTGCCACTTACCACAA	FAM189A1	1.5661	0.0047628
UQCR11_29733_GCTCCCGGTAGCGTGGGCCC	UQCR11	1.7465	0.0048903
C10orf113_70792_TTTATCCCATAGGCCTGGTA	C10orf113	1.5459	0.0049396
COX5A_24658_CAGGGGCCTTACCCACGGCG	COX5A	1.5934	0.0051406
SNAP25_17821_CAACCAGTTGCAGCATACGA	SNAP25	1.5895	0.0057178
ATP8A1_27946_GAAATACTGTCGTACTCCAA	ATP8A1	1.7417	0.0059963
APOL5_51413_GAGACAAAGCCAGCCGACTG	APOL5	1.6444	0.0064523
CPOX_3816_GGGCACGGCTCGACGTCGAG	CPOX	1.6008	0.0067699
OR10K1_71931_CTGCGCTACTCAGTGCTCAT	OR10K1	1.9264	0.0071147
TTC19_40948_CGTCTAAGCACATGCCCAAG	TTC19	1.5753	0.0070089
FGR_6320_GTGACCGAGTTCATGTGTCA	FGR	1.5546	0.0070468
MAGEB1_11044_TCATGTACCGAATCTGACGA	MAGEB1	1.756	0.0074521
ETS2_5841_CAGACACAGAATTACCCCAA	ETS2	1.6574	0.0075516
CTBP1_4178_GGATGGCCGGGACTGCACAG	CTBP1	1.5875	0.0076507
SGK223_62997_AGCTAAAGTGAGATTCGTCG	SGK223	1.7069	0.0077458
FKBP4_6362_AGGACTGCCTGCTGAACCGT	FKBP4	1.6416	0.0077644
COX5A_24660_TGAGGAGTTTGATGCTCGCT	COX5A	1.5067	0.0077828
SNAP25_17824_GTTATGTTGGATGAAC	SNAP25	1.6755	0.0078567

AAGG			
FGR_6318_AGGGGACTTCAGAAGCTAC G	FGR	1.7388	0.0079826
MAGEA10_11032_TGGTATTAGAGGAT AGCAGG	MAGEA1 0	1.5228	0.0080265
SHOX_17325_AAAAGCAAGGACGGTAA CGG	SHOX	1.542	0.0080567
C22orf24_33675_GATAAGAAATCCAAG GCTCG	C22orf24	1.775	0.0081649
TTC23_48268_CTCACATTGGAAACTAG CAG	TTC23	1.6621	0.0081627
LRRC39_59361_AATCGTTCATACTCAG ATCA	LRRC39	1.6682	0.0081826
SLC9B2_60142_GTGCACGCCTCCACAA TACA	SLC9B2	1.5076	0.0081589
OCSTAMP_59566_CTTCTACCTTCACAT GCTCA	OCSTAM P	1.4495	0.0081593
ZNF768_49751_ACTCCTACGGAGATAT TCAA	ZNF768	1.7324	0.0083195
ADRM1_29954_GTGACTCCGGATAAGC GGAA	ADRM1	1.4875	0.0083103
ADRM1_29951_ACGTGCTGAAGTTCAA GGCA	ADRM1	1.3854	0.0083413
CXorf21_50978_TCCAATAGGATGAGTA CCGC	CXorf21	1.6131	0.00848
DUSP15_59617_AGCGACCGGGATGCGA AGGT	DUSP15	1.4866	0.0084682
BTN3A1_30174_GATCATGAGAGGCAG CTCTG	BTN3A1	1.3614	0.0086302
CPOX_3813_AGATGTTGCCTAAGACCT CG	CPOX	1.365	0.0093197
ADCK5_65024_TGCCAGGATGTGGGTC CGAG	ADCK5	1.4932	0.0095875
PTRHD1_71982_CCCAGATGAGACCACC CTAA	PTRHD1	1.7295	0.010067
DMPK_4867_CTTCTACGCGGATTCCAC GG	DMPK	1.4716	0.0098496
CDH3_2838_GAATAAGCCACTGGACCG GG	CDH3	1.5103	0.0099403
OR8B3_71746_ATAGCGATCATATGCCA TTG	OR8B3	1.3312	0.010233
JARID2_9911_CTGAGCCGACCGATGCA GAG	JARID2	1.5983	0.010722
TAS2R3_37366_TGGCAAGCCAAATGCT	TAS2R3	1.4194	0.010667

CAGA			
ZNF550_63587_CTGCACAGGTAGCATGTGAG	ZNF550	1.3193	0.011138
NEUROD6_47242_CTGAGCTCACCACTCCCCCA	NEUROD6	1.5558	0.011483
KRTAP21-3_75666_TGTGGATTTGGCTCTTGCTA	KRTAP21-3	1.3403	0.012668
PAK6_44595_AGCTACGTGAAGATTGGCGA	PAK6	1.5864	0.014789
ADCK5_65023_GTGCCTCTGCCATGACATAG	ADCK5	1.2903	0.01445
ZNF711_20462_ATGGCAAGGGTACACTGTGA	ZNF711	1.5355	0.015396
GRM7_7809_GATCTCTGTGCTGACTACCG	GRM7	1.2746	0.015127
SAG_16796_TTTGCGGATCAGTAATCGCA	SAG	1.4135	0.016198
OR10K1_71932_GAAGGGCAGATGAAATACTA	OR10K1	1.4426	0.018249
CCDC153_67228_GGATGAAGCCCGTCGAGCCA	CCDC153	1.3524	0.018746
MMEL1_49135_CACACGAGTGAACTACCGCA	MMEL1	1.5199	0.019591
HIST1H2BH_21680_GCTTGTTGTAATGAGCCAGG	HIST1H2BH	1.4274	0.0195
OCSTAMP_59568_GGTGCTGAGGTGTGTCACCG	OCSTAMP	1.2686	0.019422
CYP26A1_4451_CGTAGCATTCGAGTGCCTCG	CYP26A1	1.4828	0.020262
SOCS3_23606_GCGGATCAGAAAGGTGCCGG	SOCS3	1.4143	0.020225
AGBL1_58630_GCCTACGTGCAGATCCGACG	AGBL1	1.4294	0.020776
ADNP_32678_TTGCACATCACTTACGAGAG	ADNP	1.3813	0.020966
OR6C74_66504_TGGAGACCCATCAGGAGTGG	OR6C74	1.2109	0.020933
FKBP4_6364_CCCCCTCGCCAATCTCAAAG	FKBP4	1.5452	0.022378
AXIN2_21566_GGGAGCCTAAAGGTCGTGTG	AXIN2	1.2019	0.021944
MRGPRX2_57931_TGGAACAGAAGAAGTTACTG	MRGPRX2	1.5533	0.024872
FAM26E_66434_TCGGCAGCGAGCATA	FAM26E	1.2612	0.024737

ACATG			
C10orf82_61168_GTTGACAGGTTTCAGTTTCG	C10orf82	1.2597	0.025521
POU6F2_30698_TGTGGAATCAAATGACAGCG	POU6F2	1.4211	0.026833
GRM7_7812_TTATGTGTCTACCCTCGCAT	GRM7	1.2209	0.026535
C22orf24_33678_TTGGGAGGGGCTGTCGTGCA	C22orf24	1.3259	0.027152
PAFAH2_13258_CCAGTGCAGTACTCATAGCG	PAFAH2	1.2294	0.02781
PTCHD3_70251_GTCCAATGTCTATTCAAAAG	PTCHD3	1.4868	0.03027
LOC388813_71197_AACACCAAACAATCTCTTCG	LOC388813	1.3317	0.029938
SLC10A6_69673_AGGAATGGTCAGGCACACAA	SLC10A6	1.3436	0.03016
LMNA_10705_AGTTTAAGGAGCTGAAAGCG	LMNA	1.451	0.030818
STIP1_29691_CTGAGAGTGGTCATGATCCG	STIP1	1.3197	0.031214
RILPL1_70049_ACGCGACGAGATCCGCGCCA	RILPL1	1.4242	0.032116
CLPP_21383_GCGCCTATGACATCTACTCG	CLPP	1.4184	0.032495
IAH1_68038_CGCTGCGAACCTAAAGAGCA	IAH1	1.607	0.035821
APOL5_51412_CTTGGTAAGCAACTCGTGAG	APOL5	1.2903	0.034444
CCDC153_67226_GAGCTGCTCCGAGACCACTT	CCDC153	1.2411	0.034604
ETS2_5842_GCTGTCCGCACCGTTCTCAG	ETS2	1.3492	0.035283
TAS2R3_37365_TCACCACCCTGGCACTCTTG	TAS2R3	1.1601	0.034778
TSPY2_47844_CTGCACAGCCTCCATCCTGA	TSPY2	1.4274	0.039865
DEK_21063_CGAAATGCCCGGTCCCAGAG	DEK	1.1646	0.03825
IGFL2_61896_TTGTGTCCAAGGGAAGTCAT	IGFL2	1.2268	0.038686
UBQLNL_61207_TGGTATGAATCAAGCTCCAG	UBQLNL	1.418	0.041155
KNDC1_54934_GCAGACCCTCGAGATG	KNDC1	1.1864	0.03962

CTAG			
DUSP15_59618_CACATACGCTGTCACA ATCG	DUSP15	1.1785	0.041964
HIST1H2BH_21679_GCATCTCCTCCAAA GCCATG	HIST1H2 BH	1.3189	0.045214
PRELP_14728_GTTGCGGCTATCACAGT AGA	PRELP	1.2939	0.045625
MAGEA10_11030_CAGGCTATCTGAGC ACTCTG	MAGEA1 0	1.1587	0.045734
DMRTC2_47203_ATCATCTTACCAGGG AAGGG	DMRTC2	1.1924	0.046264
UBE2O_47077_GACTTCGTGGTAGATAA GCG	UBE2O	1.3116	0.047491
BMP5_1785_AAAGGCCCAAGCTTTAGA TG	BMP5	1.1228	0.047489
LAMP5_33609_GCCGATATCGCATTGAC CCG	LAMP5	1.2486	0.048514
CLYBL_64287_TCTAGAAAGAGACCTA CTTG	CLYBL	1.3158	0.049816
ANKRD30B_70335_GAAGGCTCATCAG AGATAGT	ANKRD3 0B	1.1359	0.049444
PROSER2_66470_GACGATTGACTCCCT AGACG	PROSER2	-4.1616	0.048837
OR10H5_67866_ATGAGCACGTTGTAAC GCAG	OR10H5	1.1468	0.052646
FAM26E_66435_TTCAGTTCTTCATTGA CGGT	FAM26E	1.2449	0.054836
ADNP_32675_GTTGGTCAGTCAATGAG ACT	ADNP	1.166	0.056975
UBQLNL_61205_AGTCTTCACTATCACT CGAG	UBQLNL	1.1537	0.057566
IGFL2_61893_CTGACACATAAGCAGGA GCT	IGFL2	1.2545	0.059409
RAD18_44446_TTCTTGATCAGAGAAAT GAG	RAD18	1.1809	0.059256
DUSP15_59620_GGCAGTTCCCCCCATT AAGG	DUSP15	1.1956	0.061195
TRPM6_60982_GGAGCGTGGATAATAA CTGA	TRPM6	-3.5288	0.05881
OR10H5_67868_GACAGTGAGCAGGAA GGAGT	OR10H5	1.2388	0.06248
ADCK5_65022_CGCGGGGCACCACGAC GTAG	ADCK5	1.1132	0.061413
XCL2_18554_TCTGCTAACTGGCAGTCG	XCL2	1.2434	0.06444

CT			
ZBP1_51483_AAGCCATCCAGATTGGACAC	ZBP1	1.1596	0.06357
GRM7_7811_TGGTACTGGTAACCATCGCA	GRM7	1.1371	0.064062
CHPT1_44799_AGGATAAGTTCCTAAGCGAG	CHPT1	1.1353	0.065996
SVEP1_50531_AGTTTGTAAAGACCTAGTTG	SVEP1	1.1903	0.068124
SULT1A1_18469_CCACTTCTACCACATGGCCA	SULT1A1	1.1968	0.068241
IGFL2_61895_TGTAACAGCACTGCTCCAAG	IGFL2	-3.4894	0.073246
MRGPRX2_57929_CCGCCCCAGACACCTGTCAG	MRGPRX2	1.1814	0.076687
OR5L2_34995_AGAAATCTACAAAGGACAAG	OR5L2	1.2813	0.079009
CYP26A1_4450_CAGGTAAGTGATCAGAGATG	CYP26A1	1.156	0.077467
JARID2_9910_CCTTAGACTGGTCATTACCA	JARID2	1.0921	0.07714
UBQLNL_61208_TTGGTCATCAAGTCCAAGCA	UBQLNL	1.0627	0.078858
ZNF81_69802_CCAAATGAATCACGTTTATG	ZNF81	1.2833	0.082723
PGBD1_53785_TGGAGATGAGCTGATCCGTG	PGBD1	1.2579	0.083217
C21orf140_76364_CCCAGAAAGTCTCGTAACTG	C21orf140	1.3126	0.085514
THSD7A_65940_TTATGTGCATTAACAAGACG	THSD7A	-3.308	0.081006
GRM7_7810_TCCGGCTTGACGAAAACCGG	GRM7	1.0886	0.086719
LPCAT1_50270_TCACAACCAAGTGGAAATCG	LPCAT1	1.185	0.090541
DMPK_4865_ACACTGTCGGACATTCGGGA	DMPK	1.332	0.093932
NEUROD6_47241_AGTTGTCCAGAGCGTCGTTG	NEUROD6	1.0165	0.088277
KRTAP21-3_75665_TCAATTACAAAAGTGTGTGT	KRTAP21-3	1.0476	0.08916
PRSS1_15040_GGCGTTGATTACTGCACGTG	PRSS1	1.2772	0.095107
HOXA9_8659_GCGTTGGCCGCTATGCG	HOXA9	1.204	0.10102

CCG			
MORN1_50330_TGTCACACGTACTCACAATG	MORN1	1.2075	0.10206
OR51A7_58172_GGGGATGGAGAACCAAATGT	OR51A7	1.1509	0.10703
MAGEB1_11043_GAGGTTTAGCTTACTGACGA	MAGEB1	1.2447	0.11256
CLPP_21382_CACATGTACATCAACAGCCC	CLPP	1.1385	0.11291
MEOX2_11371_TATGGTCAGATCTTCCATGG	MEOX2	1.0432	0.11305
PTCHD3_70252_GTCCAGCAGTGAGTCGCTGT	PTCHD3	1.0599	0.11486
ALAD_579_CACACAGGTATGGTGTGAAG	ALAD	1.1447	0.11781
B4GALT5_24521_ATGGATGTGGACAGATGCCG	B4GALT5	1.0889	0.11766
TTC23_48270_TCTGTATTCTCACTATAGGG	TTC23	1.1507	0.12117
C10orf113_70789_CCACACAAGAAAAAAATGAC	C10orf113	1.1232	0.12086
TSPY2_47843_AGGCTGCGGCAGGGTTCCTG	TSPY2	1.2246	0.12631
SEMA4G_46313_GTGTCTCATACAGTGGAATG	SEMA4G	1.0397	0.12149
KRTAP15-1_66529_AAGTGGAAACTGGATACCTC	KRTAP15-1	1.0838	0.12678
PRSS1_15037_ACACTTACGACTTGTAGCAG	PRSS1	1.0039	0.12541
SH2B1_34220_AGGCGACACTTCTGCCACTG	SH2B1	-2.6669	0.12438
POR_14429_ACATGCCTCGCATCCCGTAG	POR	1.0017	0.12978
HOXA9_8658_CATTAAACCTGAACCGCTGT	HOXA9	0.98665	0.13089
OCSTAMP_59565_ATTGCTACCTGACAGACCTG	OCSTAMP	0.92231	0.13557
AGPAT5_42333_GACCCCCCAGGCCAACACGT	AGPAT5	0.96623	0.13678
C9orf163_63102_CAAATGTTAGCTGAGCAGAT	C9orf163	0.95351	0.14695
POU6F2_30695_ATGTTGGTGAGATTCGCTGT	POU6F2	1.1158	0.15485
CXorf21_50976_GACTTGTGTTTCATCCA	CXorf21	1.0167	0.15616

CAG			
EDIL3_26955_TATGTTTGTAAATGTCCCCG	EDIL3	1.2587	0.16782
JARID2_9912_TGAGATTGTGTGGTTGACAG	JARID2	1.0144	0.16222
BMP5_1787_CGAGATAACTGTATGCGACG	BMP5	-1.9488	0.15733
LRRC39_59364_TCTTTCTTACCAATCCCTGG	LRRC39	-2.2726	0.16042
NUDT3_30337_AGCAGTTCGTGAAGTCTGTG	NUDT3	0.93122	0.16592
XCL2_18553_TAGGGAGTGAAGTCTCACAT	XCL2	-2.335	0.16204
SRRM4_53761_CGGTGGCGGTGAGACTTTCG	SRRM4	0.95554	0.16875
CXorf21_50975_AGTACTGGAATGACATCCAC	CXorf21	1.0606	0.17709
OCSTAMP_59567_GCAAACAGGCAGCGGACTGG	OCSTAMP	1.1177	0.18355
PEBP1_13225_ACAGTCCTCTCCGATTATGT	PEBP1	-2.1738	0.17204
PRIMA1_61404_CGGGACTTGGTGCTGCGCCG	PRIMA1	0.99655	0.1836
OR8B3_71745_AGTGGTCTACATTGTCACCA	OR8B3	-1.8061	0.18184
PAK6_44597_GGGCACCCTTGGCAACCGTG	PAK6	1.0072	0.19179
POU6F2_30697_CATACCTGTAGAGGATTGGG	POU6F2	1.0138	0.19931
CD1A_2532_GAAGACCCAAGATGAAACGT	CD1A	1.0654	0.20558
NRG3_28971_AGGAGGGAGTAGAATCGTGA	NRG3	-1.9567	0.1975
DMRTC2_47206_GGTGTCACCGCCCATCTCAA	DMRTC2	-1.9759	0.19789
C8A_2024_GGTCTCCATTACACACAAGG	C8A	1.0267	0.22498
ZNF550_63588_GCCCGCTCAGATAAGACTGG	ZNF550	0.859	0.21762
EDIL3_26953_CCACAACACTAGAACAGTTG	EDIL3	-1.8908	0.2162
MCCC1_44588_AGAAGTTTGTAGACACACCG	MCCC1	-1.8704	0.22014
FAM109B_62325_GACTCCCCAGCGGAC	FAM109B	0.80803	0.22676

CACAT			
LMNA_10706_GCGCCGTCATGAGACCCGAC	LMNA	0.93696	0.23682
SMAD7_10978_GGGAGCGAGTAGGACGAGGG	SMAD7	-1.8596	0.22521
FAM189A1_32569_GCTCCCCCCTCCGTACGAGG	FAM189A1	0.94047	0.2479
ZNF711_20461_ACAGAGAGTGAGTACACCAG	ZNF711	0.90417	0.24633
CDHR5_39667_ATCATCTACAGCATCTTTAG	CDHR5	-1.8233	0.23937
C8orf4_44480_CAGACCAAGAATCACTAGAA	C8orf4	0.95854	0.25427
FBXO31_49951_AGACAGTGGAGATCGACCTG	FBXO31	-1.7647	0.24188
ADNP_32676_TCAGATTGTATGTAGTTACC	ADNP	0.88375	0.25689
C5orf47_60163_GAAGCCGAGGGAAGCAATGG	C5orf47	0.80507	0.25548
DEK_21064_TCTAGAAAAGAGTCTCATCG	DEK	0.90271	0.27354
MEOX2_11369_GATGTCTTCCCCACCGAGTG	MEOX2	0.79782	0.26997
TSPY2_47846_TCTGGATGACGGCGCCTCTG	TSPY2	0.91782	0.28113
OR10H4_59178_GCACATTGTAACGCAGTGGG	OR10H4	-1.5017	0.26809
RASEF_63127_ATATAAATAATATCTCACCA	RASEF	0.9643	0.29246
SEMA4G_46314_GTTGGCACTGAGAGAGAACA	SEMA4G	-1.4459	0.27203
OR10H5_67867_CGGTGATGGAGAGGGCACAC	OR10H5	-1.5463	0.27354
GPC3_7191_GACATCAATGAGTGCCTCCG	GPC3	-1.282	0.27526
NUDT3_30338_GTGTTTCCGCAGCGAGAGCG	NUDT3	0.77413	0.28554
SRRM4_53762_CTGGGAACGTCCTCGCGAGG	SRRM4	0.74122	0.28848
CT45A1_73501_GGCTTACTTACTCTTTGCTT	CT45A1	-1.3487	0.28246
TIMM44_28190_ATCTGATAGGTCGACCCATG	TIMM44	0.84818	0.29668
NAPB_47118_TACAAAAAGGCAGATCC	NAPB	-1.4148	0.28317

CCA			
SH2B1_34221_CCTCCCACCCCCAATATGCG	SH2B1	-1.5091	0.2833
OR5M11_65295_GATATTAGTCGACATCTGCG	OR5M11	-1.4587	0.28376
PTCHD3_70250_GTCACACCGACTGCCTAGAG	PTCHD3	-1.434	0.28854
THSD7A_65939_TGTGTTGAAGGACGATACTG	THSD7A	-1.4908	0.30218
C8orf4_44481_GCCCACGGCTTTCTTACGAG	C8orf4	0.84171	0.32142
OCEL1_49441_CCTGGAACACTGCGACATAG	OCEL1	-1.4128	0.30799
LMNA_10708_TCTCACTCATCCCAGACACA	LMNA	-1.9102	0.3091
EHD2_37139_ATGATGTAAGCGTGAACCTG	EHD2	0.76216	0.32196
UQCR11_29734_GGTCCCGACGGCCTACACAT	UQCR11	-1.2234	0.31537
LRRC39_59363_TCAGTTTCAGCAGAGAAGAA	LRRC39	0.82034	0.33298
AXIN2_21565_GCTCACACTCAATTCGCGGG	AXIN2	0.76443	0.32859
FBXO31_49954_TCCACACCGACACCATCTGG	FBXO31	0.75844	0.3314
MSI2_58767_GCCAAGCATGAACGCGTCCA	MSI2	0.69035	0.32853
ZNF768_49752_GAGCCCTTCGAGGTCCAGGT	ZNF768	0.8271	0.34049
LAMP5_33608_ACTGACTGCGTCTTTGAAGT	LAMP5	0.6895	0.33682
WASF2_27218_GAAGCAGAGTCTGACTGTGG	WASF2	-1.2973	0.33831
ZCCHC3_54836_CCTGTTCCTACGCGTCTACG	ZCCHC3	-1.3347	0.34256
SAG_16795_TTTCTAGATCTATTTCCATG	SAG	-1.295	0.34847
PEX11B_23013_CACTTCTCCTGATCCACACG	PEX11B	-1.2383	0.34958
UBQLNL_61206_CCAGGTGGGAATAATGCCCT	UBQLNL	0.69073	0.36196
ZNF81_69804_TTACCCAAAAATCAACACTC	ZNF81	0.74818	0.36783
STIP1_29692_GTCGACCTAAAGCCTGA	STIP1	-1.0677	0.35907

CTG			
UHRF1BP1L_31649_TCTGAGCCTTTGACAAATGG	UHRF1BP1L	-1.233	0.36227
PRSS1_15039_GATGAGGCACTTCGTGCCAG	PRSS1	0.71082	0.38219
LAMP5_33607_ACTCATAGGACTTCCCAGCG	LAMP5	0.67951	0.38554
ANKRD52_67332_GTCAACCAGCCGAATGACAA	ANKRD52	-1.192	0.37889
USP25_36580_GAGACTGAAAGATTACCTCA	USP25	-1.155	0.38128
HIST1H2BH_21678_GAAGCGTAAACGCAGCCGCA	HIST1H2BH	-0.96337	0.38139
EHD2_37140_CTTTGTGGCCGTCATGCACG	EHD2	0.76954	0.40712
COX5A_24659_GATGCCTGGGAATTGCGTAA	COX5A	0.70291	0.40344
SYT10_69316_TTCTAGACATGACATGATTG	SYT10	0.74046	0.41614
KNDC1_54931_CGACGTCCACAACTACGTGA	KNDC1	0.6673	0.41016
SLC10A6_69674_AGTCCCACAGCAATGCCCCA	SLC10A6	0.5995	0.40564
UNC80_68046_CTGACCAGACTGACCCGCGG	UNC80	-0.99539	0.4025
CCDC153_67227_GCCTTCGGGGGCAGCTAGGT	CCDC153	-0.98845	0.40415
FKBP4_6361_AGCACCATAGTGAAAGAGCG	FKBP4	0.7437	0.43135
RILPL1_70050_CCCACCCAGGCATGTCAGAG	RILPL1	0.70892	0.42869
PRELP_14726_CAGTCAGGGAAGATAGATGG	PRELP	-0.99889	0.41017
C9_2037_CTACCGAAGACCTTGGAACG	C9	0.60319	0.42118
SULT1A1_18472_GTGCTGGTACCAGGATCCGT	SULT1A1	-0.94059	0.41786
TWIST1_19757_AGCGGGTCATGGCCAACGTG	TWIST1	0.66084	0.4384
ZBP1_51485_GGACGATTTACCGCCCAGGT	ZBP1	0.63855	0.43624
CT47A10_74533_TGAGTTGACGGTTCTCTATG	CT47A10	0.62282	0.44069
C9_2040_GATGCATAAAGATGCGACTT	C9	0.62277	0.44721
PHKG1_13876_TCCGAGTAATCATCCCA	PHKG1	0.60742	0.44689

CTC			
ADCK5_65021_CACCCCTCGAAGGACA ACAT	ADCK5	0.608	0.45594
CTBP1_4180_TGATGCCCAAGGTCTCCC CG	CTBP1	-0.95149	0.4434
KRTAP15-1_66532_CTTATGTAGTTAGT CTGGCG	KRTAP15 -1	-0.87723	0.44652
CHPT1_44800_CATTTGGAGGAGCAACA ATG	CHPT1	-0.8789	0.45317
C5orf47_60162_CGGGCATCGAGAGTTC AGAG	C5orf47	0.55697	0.47384
IAH1_68040_TTTCAGGTTACAATACCA GG	IAH1	-0.92835	0.46523
ANKRD52_67329_CTGGACGTAAGGGA CCACAA	ANKRD5 2	-0.81798	0.47008
OR10K1_71930_CATATAGCGATCATAG CCCA	OR10K1	0.57885	0.48628
C10orf82_61166_CTGCAGATTGTACTGG TGCA	C10orf82	0.51584	0.49451
TTC23_48269_TCAGGGAGACACGAGCA CCA	TTC23	0.56585	0.50418
KLHL18_32315_GCTGTGCGACGTGACC CTCA	KLHL18	-0.81824	0.49075
ABCC1_11743_AGTACACGGAAAGCTT GACC	ABCC1	0.7111	0.53945
PEBP1_13227_GACATAGCGGTGGAGGC CTG	PEBP1	0.58497	0.51741
JARID2_9909_ACCGACTCGATGTAGAT GAG	JARID2	0.55675	0.53582
ZDHHC5_34087_AGCTCCCCTTTACAAA ACAG	ZDHHC5	-0.78575	0.51687
ADRM1_29953_GCTTGAACTCACAGTC GTCA	ADRM1	0.52456	0.53317
CYP26A1_4452_GGGAAGCCCATAGTCC CGGG	CYP26A1	0.6232	0.55706
TSPY2_47845_GCTGTTGGATGACATAA TGG	TSPY2	0.68856	0.5714
TUBAL3_50171_GCTGCTAACAATTACG CGCG	TUBAL3	0.64613	0.56557
CLPP_21384_GGAAAGTGAGCGGCGAG GCG	CLPP	0.57018	0.55676
ANKRD30B_70334_ACTTTCTGATACAT AGACTC	ANKRD3 0B	-0.71303	0.53129
CLYBL_64285_AAGGACCCGGGATTGC	CLYBL	0.52781	0.55625

AAAA			
SNAP25_17823_GGGCAATAATCAGGAC GGAG	SNAP25	-0.71581	0.54096
PRRG3_48826_TGGCATCTGAGCTCTGC GAG	PRRG3	0.52451	0.56318
PAFAH2_13259_GATCCCTTTCCGTCGA GTTG	PAFAH2	-0.61211	0.54694
ZNF549_66697_ACAAGAATGAGCATTT GGGA	ZNF549	-0.69422	0.54766
TAS2R3_37363_CAGAGATGCGGTACTA CCAC	TAS2R3	-0.63933	0.54778
OR51A7_58169_AGACATAATTAGAAGT ACTG	OR51A7	-0.69523	0.55305
NEUROD6_47240_ACTGGCCAAAAACT ACATCT	NEUROD 6	0.51762	0.58093
RAD18_44443_AGTGGATTGTCCTGTTT GCG	RAD18	0.48068	0.5763
OR5L2_34996_GAGCAAGCAAGACTTA GGAG	OR5L2	0.49155	0.58128
ZNF550_63586_AGATGATGGTCTGCAC TCCA	ZNF550	-0.55088	0.56881
USP25_36581_GGCACCAAGGCACATAA CGG	USP25	0.4707	0.58582
UPB1_39391_ACGCATAAAGGCTATCGT AG	UPB1	-0.65295	0.57014
KNDC1_54933_CTCTGCCACGTACTCGA GGG	KNDC1	-0.62773	0.57473
PGBD1_53784_CTGTCCCTGAATATAGA CAG	PGBD1	0.52921	0.60454
USP25_36582_TGTGATGGTATGGAACC ACT	USP25	0.4648	0.59241
TUBAL3_50172_GTGGTGAGGACAGAG TTATA	TUBAL3	0.50938	0.60243
KIAA1024_32232_AGTGGGCCCAAAAT ATGTAG	KIAA102 4	-0.61496	0.58727
DEK_21062_CAAAGCCTTCTGGCAAAG TG	DEK	-0.56615	0.58827
SERTM1_72177_AGATGTAGACGTTTGA CAGG	SERTM1	0.56586	0.6443
NRG3_28972_CATGCTTCTCAAATGGAT CG	NRG3	0.43719	0.61767
MMEL1_49136_CCAGAACATGGACCCG ACCA	MMEL1	0.49078	0.64361
MORN1_50327_CTCTCCCAGAACAAGC	MORN1	0.57558	0.67021

TACG			
UBE2O_47075_ACTTAAAGCGCAAACA CAAG	UBE2O	0.60196	0.67845
OR7G2_71861_AATCGGAGCATCACGTA CTC	OR7G2	0.46014	0.64541
PPP6R3_42225_GTTACATGGGACACCT AACG	PPP6R3	0.50794	0.67164
MMEL1_49138_GACAGGTGGAACGAGA CCGT	MMEL1	-0.50993	0.63058
FGR_6317_AGCTTGGATTGAGTCAACA G	FGR	-0.50247	0.63285
RASEF_63125_AAGTGGAATACAAGCA CCAG	RASEF	0.39431	0.66013
ZNF549_66698_AGAGTATGCACATAGA AGCA	ZNF549	0.51627	0.69351
PRELP_14725_CAGGTAGAGCTGGTGAA TGG	PRELP	0.39798	0.66891
ATP8A1_27948_TGTCCTAGCACCGTTC CACT	ATP8A1	-0.47265	0.65211
OR51A7_58171_CCTCAACATGGTAGGA AGGG	OR51A7	-0.47854	0.65357
C9orf163_63103_CCGCCCGATGGCATCG CGGG	C9orf163	0.35224	0.67187
NAPB_47116_CATCGACATTTACACAG ACA	NAPB	0.42518	0.68978
MAGEB1_11041_AGCGGCGAGAGGCTC CATTG	MAGEB1	0.42988	0.693
CXXC1_37104_AGAAGGAGGAGCGATA CAAG	CXXC1	0.45231	0.70572
UBE2O_47076_ATGTAGTCCCCATACAT GAA	UBE2O	-0.43798	0.67265
FAM109B_62327_TCTCCAACTCGCGTA CCACC	FAM109B	-0.94075	0.68027
SMAD7_10980_TCTCACCTAGTTCGCAG AGT	SMAD7	-0.94075	0.68027
TIMM44_28192_TCTCGGCCGGAAAATC AAGG	TIMM44	-0.94075	0.68027
TUBAL3_50173_TCTCGGATAAGGTACC AGGT	TUBAL3	-0.94075	0.68027
OR10H4_59180_TCTGTGTTCAATCCAG ATTG	OR10H4	0.49408	0.76491
SVEP1_50532_ATATTCCGTGTACATCC CAG	SVEP1	-0.39475	0.69377
BTN3A1_30171_AATTGTTCCCTACCTG	BTN3A1	0.33736	0.71266

AGTG			
CYLD_4331_TATGGGGTAATCCGTTGGAT	CYLD	-0.38786	0.69657
PEX11B_23015_GGCGAATCTCATAAGCATCA	PEX11B	-0.39266	0.69836
FAM189A1_32570_GGCGCTCGGATGCCTCATCG	FAM189A1	-0.39212	0.69855
CYLD_4329_AAAGGCCTCCAAATAGACGT	CYLD	-0.3866	0.70145
DMPK_4868_TCGAAATCCGGTGTAAAGGG	DMPK	-0.37014	0.70795
LOC388813_71200_TGTGAATGACAAAATCTGGC	LOC388813	0.44181	0.76826
RDX_15975_ATGATAGACTCCTACCCCAG	RDX	-0.36492	0.70927
SLC9B2_60144_TGTCCAGTAAACCATGTGGA	SLC9B2	-0.36911	0.70973
UPB1_39393_CAGAGTGGGTGATTTCAACG	UPB1	0.38006	0.7469
BHLHE41_49186_GGTGTCCGTGTCGTTCTCGG	BHLHE41	-0.36491	0.71372
RAD18_44444_GACAGCAAGATTCTTTGAAG	RAD18	0.31	0.73135
TAS2R4_37369_CACATCATTTAATATCAGTG	TAS2R4	-0.35879	0.71723
FOXE3_6403_CGGGCGCGTAGGGCGCGTAG	FOXE3	0.28108	0.72939
WASF2_27217_AGCCAGCCATTACCTGTAAG	WASF2	-0.32308	0.72855
OR7G2_71862_AGTCAGAGATGACAGCCAAG	OR7G2	0.37048	0.77118
MRGPRX2_57932_TTGATTTCATCACTGCAGCG	MRGPRX2	-0.32122	0.73719
ALAD_580_GGACATGATGGATGGACGCG	ALAD	0.32416	0.77
SULT1A1_18470_GGAGACTCTGAAAGACACAC	SULT1A1	0.33831	0.78368
UPB1_39392_AGAATTTGCTGAGTCAGCAG	UPB1	-0.30653	0.74803
ZNF81_69801_ACACATTTGCTAAGCTCATG	ZNF81	0.3445	0.79522
PTRHD1_71983_GCTGGGGCGCATGCGCAAAG	PTRHD1	0.40985	0.82571
GLDC_7206_AGGGTAACTTCAGCTCAG	GLDC	-0.29443	0.75514

TG			
PTRHD1_71984_GGCTTGTCACGCGGCCACCG	PTRHD1	-0.2905	0.75646
RASEF_63126_AGGCTGCAGAACATAAGACA	RASEF	0.29981	0.78475
C10orf82_61165_AGAAACCTGCCAATCACACC	C10orf82	-0.28817	0.76078
PRIMA1_61403_AGTGAGCCATTACTTACCTG	PRIMA1	-0.28473	0.76325
AGBL1_58631_GGATATTTGAGGATATTCGG	AGBL1	-0.20553	0.77565
SYT10_69314_CCTTCCGAAGACACCTGCCG	SYT10	-0.24219	0.77693
BTN3A1_30173_CCTTCTTCAGGAGCGCCCAG	BTN3A1	-0.25346	0.77794
FOXE3_6404_GCTCCTGCGAGCGGCGACGG	FOXE3	0.2545	0.7989
AXIN2_21567_GGGAGTGGTACTGCGAATGG	AXIN2	-0.25042	0.77916
AGPAT5_42331_AAAATCACAAGATACATCTG	AGPAT5	0.24561	0.80198
HOXA9_8657_ACCACAAGCATAGTCAGTCA	HOXA9	-0.25236	0.7839
TAS2R4_37368_AGTGGTCAATTGCAAAACTT	TAS2R4	-0.2384	0.79116
KIAA1024_32231_ACTTGGACGACTGCCTGATG	KIAA1024	0.25534	0.82804
ARVCF_1230_CACCTGGCGTACTGTCCGAG	ARVCF	-0.21886	0.80618
SRRM4_53759_AAGAGCCGGGAGCTCAACAG	SRRM4	0.20718	0.82572
DUSP15_59619_GCTCACCAATGAAGTTTCCG	DUSP15	0.24639	0.8469
OCEL1_49440_CCGGGAGCCGCGAGTCTTTG	OCEL1	0.27666	0.8668
RDX_15973_AGTACAACAGATGAAGGCTC	RDX	0.21309	0.83539
FBXO31_49952_ATCAGGTGGATCCTGAACAG	FBXO31	0.26239	0.86471
CDKL5_18416_TTGCGGAGCCCAGTACCAGT	CDKL5	-0.19709	0.82084
PRIMA1_61402_ACCTGCACGAAGCCCCAGAG	PRIMA1	0.21253	0.85105
FAM109B_62328_TTCTCCTTTGAGAGT	FAM109B	0.22658	0.85965

CGCGA			
SLC10A6_69676_TATGACAACCTGTTCC ACCG	SLC10A6	-0.19037	0.82639
UHRF1BP1L_31647_CAAACAGACGTAT TACTGGA	UHRF1BP 1L	0.21974	0.87283
C8orf4_44482_TGGGCTGACTCGTAGCG ACG	C8orf4	0.19372	0.8653
PEX14_13699_GTACTTACCAGCGGCTC TCG	PEX14	-0.16522	0.84361
APOL5_51411_CTTCGAAAGGTAATCTA CGG	APOL5	0.19911	0.88404
TRPM6_60981_AGGTCATGATGTAGCG ATAG	TRPM6	-0.15179	0.854
PAK6_44598_TCTGAGCGCACTGACCCC CA	PAK6	-0.14273	0.85856
TWIST1_19759_CGGGAGTCCGCAGTCT TACG	TWIST1	0.17959	0.90182
LPCAT1_50267_CTCGAACGTGTAGTCA GTCA	LPCAT1	-0.10969	0.86819
GPC3_7192_GGAGCGGACTTGGTGACA GG	GPC3	0.22516	0.95327
LOC388813_71198_GCTGACAGGGCCTT CTGATG	LOC3888 13	0.1483	0.90815
ZCCHC3_54838_TGACCGCGTAGATGTC GCTC	ZCCHC3	-0.05747 1	0.88522
POR_14431_GTGGTCCCCAGATTCATAC C	POR	-0.09813 3	0.89458
C21orf140_76365_CTATGGAAAGTGTGT CATCG	C21orf140	0.14073	0.93993
SGK223_62999_GGGAGCAGTAATCACT CTCG	SGK223	0.19612	0.98334
CDHR5_39668_CCTGGATCCGAAATGC AAAG	CDHR5	-0.00313 09	0.90944
C9_2038_CTTACCATTTGTCTGAGACA	C9	-0.05306 3	0.91062
EHD2_37141_GGCACCCGACAGGATAC CCG	EHD2	-0.07028 6	0.91426
FAM26E_66433_GGGTAAGCCCAAAGA GTGCT	FAM26E	-0.06951 9	0.91869
PPP6R3_42223_CAACCTAATGACATTC AACC	PPP6R3	-0.03904 5	0.92154
NUDT3_30336_AGAACCAGGAGAGGAA GCAC	NUDT3	-0.04692 8	0.9268
SLC9B2_60141_GCACAGACAACCCCAA	SLC9B2	-0.04976	0.93226

CACA		1	
MSI2_58765_AAAACTACCAACAGGCACAG	MSI2	0.085385	0.96305
PRRG3_48825_TACCGGTTCTGAGCATAGGA	PRRG3	-0.014507	0.93568
LPCAT1_50269_GCGTCGAAGTAGGACGAGTG	LPCAT1	0.0804	0.96136
KRTAP21-3_75664_ATAACCGTTACAGCCACAGT	KRTAP21-3	-0.050919	0.93721
TWIST1_19760_GCGCACCCAGTCGCTGAACG	TWIST1	0.099465	0.99471
SOCS3_23605_ACCTACTGAACCCTCCTCCG	SOCS3	0.095491	0.989
CXXC1_37103_AAACGGTCAGCCCGCATGTG	CXXC1	-0.047529	0.93906
OR6C74_66503_CTAAAAATGAGAAATTTCGG	OR6C74	-0.0058	0.93918
SHOX_17327_CCTCGCGCATGAAGGCGTCG	SHOX	0.079006	0.99853
MAGEA10_11029_AATGAAGGAGCCGATCACAA	MAGEA10	-0.027937	0.95045
SHOX_17326_CAGCCGCACCAACTTCACGC	SHOX	-0.0079968	0.95327
PHKG1_13873_AAGGTCATCGACGTCACCGG	PHKG1	-0.0020691	0.95327
ABCC1_11744_TCTGCTTCGTCAGTGGCATG	ABCC1	0.059008	0.97863
CDKL5_18414_AAGAATGATATTGTCCATCG	CDKL5	0.051196	0.97643
C8A_2023_GGAGATTCCAGTATCTGCCA	C8A	0.066464	0.97892
TIMM44_28189_AGTGTTTGAGCCAAACGAGT	TIMM44	0.07085	0.96374
PEX14_13698_CTCTTCATCTGTCAGCCCTG	PEX14	-0.019492	0.96311
CD1A_2529_AATTCCCTCAAATGACCGAA	CD1A	0.05948	0.97848
ZDHHC5_34088_CAGACCTGAGCCGTTACACA	ZDHHC5	0.050943	0.99781
CXorf65_63235_TAGGCAACTTTACTTTACTG	CXorf65	0.039153	0.99776
BHLHE41_49185_GATCAACCACTTGCACGCCG	BHLHE41	0.048017	0.96507
TTC19_40949_CTATCAGACTGATAACA	TTC19	0.039298	0.97208

AGA			
ARVCF_1232_GTCACTAGGCTGTTACC TGG	ARVCF	0.027174	0.9934
MSI2_58768_TTACCTGGAGCGTTTCGT AG	MSI2	0.022189	0.99147
ZNF768_49753_GCGAAGGCCTTGCCGC AACG	ZNF768	0.003534 4	0.98025
